# Prevalence of myopia in Europe: a systematic review and meta-analysis of data from 14 countries

**DOI:** 10.1016/j.lanepe.2025.101319

**Published:** 2025-05-22

**Authors:** André Moreira-Rosário, Carla Lanca, Andrzej Grzybowski

**Affiliations:** a NOVA Medical School, Faculdade de Ciências Médicas, Universidade NOVA de Lisboa, Lisbon, Portugal; b Comprehensive Health Research Centre (CHRC), NOVA Medical School, Faculdade de Ciências Médicas, Universidade NOVA de Lisboa, Lisbon, Portugal; c Division of Science, New York University Abu Dhabi, Abu Dhabi, United Arab Emirates; d Comprehensive Health Research Centre (CHRC), Escola Nacional de Saúde Pública, Universidade NOVA de Lisboa, Lisbon, Portugal; e Department of Ophthalmology, University of Warmia and Mazury, Olsztyn, Poland; f Institute for Research in Ophthalmology, Foundation for Ophthalmology Development, Poznan, Poland

**Keywords:** Myopia, Prevalence, Europe, Cycloplegic refraction, Meta-analysis

## Abstract

**Background:**

Although myopia prevalence increased in East Asian countries, the burden of myopia in Europe is less known. We performed a systematic review and meta-analysis to estimate the prevalence of myopia in Europe and at the country level.

**Methods:**

We searched PubMed, Scopus and Web of Science to identify studies on myopia prevalence published until January 2024, regardless of language. We included European cross-sectional and cohort studies with defined sampling strategies and excluded clinical surveys, myopia registries, self-reported near-sightedness, and non-representative populations. Pooled prevalence was estimated using random-effects models. Heterogeneity was assessed using the Cochran's Q (χ^2^ test) and the I^2^ statistic. The study protocol was preregistered in PROSPERO (CRD42023471527).

**Findings:**

We screened 2074 records and included 22 studies (from 14 European countries; n = 128,012) in the meta-analyses. The pooled prevalence of myopia was 23.5% (95% CI: 18.5–29.3; I^2^ = 99.7%), ranging from 11.9% in Finland to 49.7% in Sweden. In cycloplegic studies, myopia prevalence was 18.9% (95% CI: 13.2–26.5%; I^2^ = 99.7%) vs. 31.2% (95% CI: 24.9–38.3%; I^2^ = 99.3%) in non-cycloplegic studies. Subgroup and meta-regression analyses exploring sources of heterogeneity showed a lower prevalence in children (6–11 years; 5.5%) compared with adolescents (12–17 years; 25.2%) and adults (18–39 years; 24.3%) in cycloplegic studies. No significant differences in prevalence were observed between sexes. Myopia prevalence increased significantly between 2000–2010 and 2011–2022 (p = 0.040), although age-specific trends remained stable.

**Interpretation:**

Myopia prevalence in Europe is lower than in Asia, with a less pronounced increase that disappears after stratifying by cycloplegic refraction and age. These findings highlight the need for age-specific data and cycloplegic refraction in future studies to reduce heterogeneity. Uneven country representation may limit the generalisability of these results.

**Funding:**

The present publication was funded by Fundação Ciência e Tecnologia, IP national support through UID/04923—Comprehensive Health Research Centre.

**Research in context**

**Evidence before this study**

Although a previous study has predicted that myopia will affect about 50% of the world population by 2050, it remains unknown if Europe will follow the same pattern of increased prevalence as reported in East Asian countries. To address this evidence gap, we conducted a comprehensive search in PubMed, Scopus, and Web of Science from database inception to January 16, 2024. The search strategy combined MeSH terms and free-text keywords related to myopia, refractive errors, and prevalence, alongside a complete list of European countries and related terms. The strategy was initially developed for PubMed and subsequently adapted for Scopus and Web of Science. Detailed search strategies are available in Supplementary Tables S1 and S2 We did not identify any recent systematic review with meta-analysis reporting the prevalence of myopia in Europe by diagnostic method. We therefore synthesised data from 22 studies published between 1984 and 2024 that met the eligibility criteria and reported pooled prevalence estimates of myopia in Europe, identifying differences in measurement accuracy.

**Added value of this study**

Our results suggest that the prevalence of myopia is lower in Europe (23.5%) than in Asia, with a less pronounced increase that disappears after stratification by cycloplegic refraction and age. We observed considerable variability across European countries, with prevalence ranging from 11.9% in Finland to 49.7% in Sweden. Myopia prevalence was age-dependent and appeared to be higher in school settings. These findings highlight the importance of studies incorporating age-specific data and employing cycloplegic refraction, as non-cycloplegic assessments tend to overestimate prevalence, particularly in younger age groups. However, significant heterogeneity remained unexplained, likely due to uncontrolled methodological biases and confounders in the included studies.

**Implications of all the available evidence**

Our findings call for further research prioritising methodological consistency, including the use of cycloplegic refraction and reporting prevalence using individual participant data or age stratification to facilitate accurate cross-country comparisons. The unexplained heterogeneity suggests that unmeasured factors such as lifestyle and genetics may also play a critical role. Addressing these gaps through well-designed population-based longitudinal studies will provide more robust data on myopia prevalence.

## Introduction

Excessive axial length of the eye that develops during the childhood period leads to an eye condition called myopia. It has become one of the most common eye health challenges of modern societies with an epidemic in several countries in East and Southeast Asia.^1^, ^2^, ^3^ While treatments to reduce myopia progression are continually expanding and becoming more effective, myopia remains a chronic condition, with progression to high myopia being a significant concern. Myopia is an important cause of visual impairment and blindness across many countries.^4^, ^5^, ^6^ In addition, vision loss has been identified as a new modifiable risk factor for dementia by the 2024 Lancet Commission.^7^ Thus, prevention of visual impairment is important to ensure better and broader health outcomes to the populations.

While heritability explains between 5% and 35% of the variation in refractive error, several lifestyle risk factors associated with modern societies have emerged as major contributors to the increase in myopia prevalence.^8^ These factors include increased education, increased near work and reduced time outdoors.^9^^,^^10^

A previous meta-analysis of 15 population-based studies from the European Eye Epidemiology Consortium published in 2015 showed that myopia prevalence increased in Western and Northern Europe.^11^ Data on prevalence of myopia in Europe are important for development of public health policies, but a comprehensive analysis of myopia prevalence is lacking. Estimates of patterns and trends in myopia derived from available data might be useful to set policy priorities. This systematic review and meta-analysis evaluates studies estimating myopia prevalence over time, focusing on cross-sectional and cohort studies across various factors, including sex, age, geographical region, diagnostic method, recruitment site (population-based vs. school-based), and urban-rural residence.

## Methods

### Search strategy and selection criteria

This systematic review and meta-analysis followed the framework of the Preferred Reporting Items for Systematic Reviews and Meta-Analyses (PRISMA 2020) and the Meta-Analyses of Observational Studies in Epidemiology (MOOSE).^12^^,^^13^ The review protocol was registered at the International Prospective Register of Systematic Reviews (PROSPERO) and is accessible online (CRD42023471527).

In the initial phase of this systematic review, we conducted a scoping search using Google Scholar to pinpoint relevant articles and identify key search terms pertaining to the prevalence of myopia in Europe. Subsequently, an extensive search for pertinent literature was conducted across multiple databases, including PubMed, Scopus, and Web of Science, to identify studies published between January 1, 1900, and January 16, 2024. A combination of medical subject headings (MeSH) and specific text words related to myopia and refractive error was employed. The search strategy initially devised for PubMed was adapted for use in the other databases, as detailed in the Supplementary Tables S1 and S2 The systematic search was performed without restrictions on publication year or language. Furthermore, a meticulous examination of references cited in the included studies was conducted to uncover other potential articles, enhancing the comprehensiveness of this review.

We included cross-sectional and cohort studies with clearly defined sampling strategies of European population-based studies or national/multinational school-based studies reporting the prevalence of myopia or providing sufficient data to compute the prevalence estimate. To be eligible for inclusion, studies needed to represent populations of any age from a European country or a specific area within a country. Additionally, studies had to provide a clear definition of myopia, including spherical equivalent refractive error (SE) of ≤−0.50 D. Given the limited data available, we extracted and analysed high myopia prevalence based on the definitions provided in the original studies. Only two studies reported the prevalence of high myopia, both using a cutoff of ≤−5.00 D. We excluded clinical-based or hospital-based surveys, myopia registries, self-reported near-sightedness, and studies lacking information on the number of eligible participants. Moreover, studies that focused on specific populations that could not be generalised to the general population, studies with non-representative populations (e.g., studies limited to males, military populations, or non-random samples) were also excluded. Additionally, studies that used visual acuity as a surrogate measurement for refractive error were not included. Furthermore, we excluded studies involving participants with pre-existing serious medical conditions, such as leukaemia, heart disorders, or syndromes associated with myopia, as well as studies involving participants with any eye disorders, such as congenital cataract. Studies that contained data solely on children undergoing myopia control treatments were excluded.

### Procedures

The study selection process for the systematic review underwent a rigorous assessment for eligibility, which was conducted independently by two investigators (AM-R and CL) in duplicate. All articles resulting from the search were included in the initial screening, and duplicates were removed. The evaluation process was facilitated using the Rayyan software, where two independent researchers, blinded to each other's assessments, performed an initial review based on the titles and abstracts of the studies. If the abstracts did not contain sufficient information to determine eligibility based on the inclusion and exclusion criteria, the full texts were evaluated. Subsequently, the same reviewers independently assessed the full text of the studies identified as eligible from the initial screening. In cases where disagreements arose between the reviewers, a third researcher (AG) was involved to resolve the discrepancies and reach a consensus.

Data extraction was conducted independently by two reviewers (AM-R and CL) using a preconceived and standardised data extraction form. The following information was collected for each study: the country of origin, the timeframe of participants' recruitment, the method used to diagnose myopia (cycloplegic refraction and non-cycloplegic refraction), the criteria used to define myopia, the number of participants with complete data, participants' age (children aged 6**–**11 years and 12**–**17 years; adults aged 18**–**39 years and ≥40 years), the proportion of males and females (sex) and the overall prevalence of myopia and high myopia. Additionally, data on study design, study site (population-based or school-based), recruitment location (urban and/or rural), sampling method, and enrolment rates were gathered.

The risk of bias in individual studies was evaluated by two independent reviewers (AM-R and CL) using the Joanna Briggs Institute Critical Appraisal Checklist for Studies Reporting Prevalence Data.^14^ This checklist was specifically designed to assess the methodological quality of prevalence studies and comprises nine questions covering various aspects of study design, sampling, data analysis, and reporting. Response rates <70% were considered inadequate.^15^ Whenever discrepancies arose between the reviewers, they were resolved through discussion until a consensus was reached. Detailed information on the quality of each study can be found in Supplementary Figure S1.

### Statistical analysis

The statistical analyses were conducted using R software (version 4.1.3) with the *meta* package. To address potential heterogeneity in the data, a random-effects model was applied, with the random-effects variance estimated using the restricted maximum likelihood (REML) method. Meta-analyses were performed using the Freeman-Tukey double arcsine transformations. However, to mitigate issues arising from extreme sample size patterns, we also employed the generalised linear mixed model (GLMM), which accounts for the binomial structure of the data and is not affected by such problems. The outcome variable was the prevalence of myopia, with the study-level effect as the predictor. A random intercept was included to account for between-study variability, capturing both true heterogeneity and random sampling variation. Confidence intervals for individual studies were calculated using the Clopper-Pearson method. To ensure robustness, we compared the results between the Freeman-Tukey double arcsine transformations and GLMM methods through a sensitivity analysis. Given the concordance of results obtained from both methods, the GLMM method is presented for simplicity and consistency in the analysis. Prevalence estimates were reported with 95% confidence intervals (CI) and prediction intervals (PI). The CI indicates the range within which the true prevalence is expected to lie, based on the studies included. The PI, derived from the GLMM, accounts for both within-study and between-study variability, providing a range within which future estimates of myopia prevalence are likely to fall. A significance level of p < 0.05 was used to determine statistical significance for all outcomes. To assess statistical heterogeneity in myopia prevalence estimates across studies, we used the χ^2^ test on Cochran's Q statistic and quantified heterogeneity using the I^2^ statistic. The I^2^ statistic estimates the percentage of total variation across studies that is attributable to true between-study differences rather than chance. An I^2^ value greater than 50% or a p-value less than 0.10 was considered indicative of substantial heterogeneity. After calculating the crude overall prevalence, we conducted a sensitivity analysis including only studies with a low risk of bias to evaluate the robustness of the findings. Subgroup analyses were conducted for predefined variables, including sex, age (6**–**11 years, 12**–**17 years for children; 18**–**39 years, ≥40 years for adults), year of study recruitment, study site (population-based or school-based), diagnostic method (cycloplegic vs. non-cycloplegic refraction), urban-rural residence, countries, and regions (based on Global Burden of Disease regions, including Northern Europe, Central Europe, Eastern Europe, and Western Europe),^16^^,^^17^ assuming an independent τ^2^. The subgroup analysis by age category was based on data extracted from studies that reported prevalences within each age category. The same approach was applied to the subgroup analysis by sex, where data from studies reporting prevalences within each sex category were included. To further explore potential explanatory factors for the prevalence estimate, univariable meta-regression analyses were conducted using the GLMM, considering variables such as diagnostic method, country, and recruitment period. Additionally, we employed funnel plots to assess publication bias and Egger's linear regression test for the Freeman-Tukey double arcsine transformations, and Peters' regression test for the GLMM. These steps were taken to ensure the reliability and validity of our meta-analysis findings.

### Role of the funding source

The funder of the study had no role in study design, data collection, data analysis, data interpretation, or writing of the report.

## Results

**Fig. 1:**
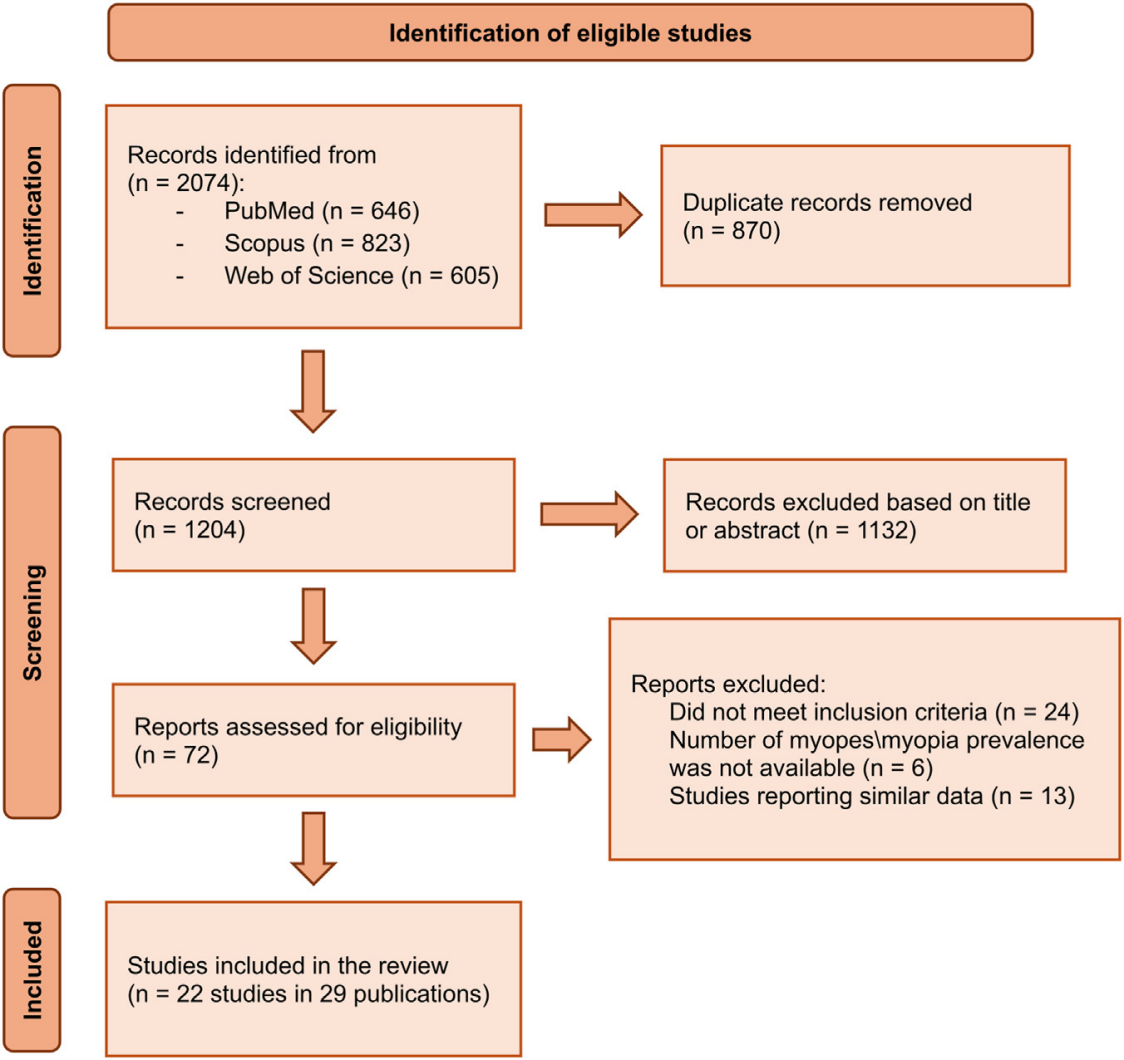
**Preferred Reporting Items for Systematic Reviews and Meta-Analyses (PRISMA) flow chart describing the study selection process**.

The database search identified 2074 records (Fig. 1). After removing duplicates, screening titles and abstracts, and reviewing 72 full text, 29 records reporting on 22 studies met inclusion criteria.^18^, ^19^, ^20^, ^21^, ^22^, ^23^, ^24^, ^25^, ^26^, ^27^, ^28^, ^29^, ^30^, ^31^, ^32^, ^33^, ^34^, ^35^, ^36^, ^37^, ^38^, ^39^, ^40^, ^41^, ^42^, ^43^, ^44^, ^45^, ^46^ A total of 128,012 individuals from 14 countries were included.

Table 1 presents the overall study characteristics. In the quality analysis, we found that only two studies were free of bias in all domains of the risk assessment (Supplementary Figure S1).^24^^,^^39^ The remaining 20 studies had problems with one or more of the checklist items. A total of 15 studies did not provide adequate descriptions of the study subjects and settings.^18^, ^19^, ^20^, ^21^, ^22^, ^23^^,^^29^, ^30^, ^31^, ^32^, ^33^^,^^37^^,^^38^^,^^40^^,^^42^, ^43^, ^44^, ^45^, ^46^ Furthermore, ten studies were found to have employed refraction without cycloplegia or did not report whether cycloplegia was used to measure myopia.^20^^,^^21^^,^^25^, ^26^, ^27^, ^28^^,^^38^^,^^41^^,^^43^^,^^46^ Fourteen studies did not adequately address low response rates or did not report the response rates.^19^, ^20^, ^21^^,^^24^^,^^26^, ^27^, ^28^^,^^30^^,^^31^^,^^34^, ^35^, ^36^, ^37^, ^38^^,^^40^, ^41^, ^42^, ^43^^,^^46^

**Table 1:** Characteristics of 22 studies included in the systematic review and meta-analysis.

Study characteristics									% Prevalence (95% CI)		
Country	Citation(s)	Location (Survey)	Period	Setting	Response rate	Sample size	Age range (years)	Diagnostic method	Overall	By country	Heterogeneity
Armenia	Giloyan et al., 2017	Gegharkunik Province	2011	School	86.7	1260	5–19	Cycloplegic	18.13 (15.89–20.55)	18.13 (15.96–20.53)	–
Bulgaria	Dragomirova et al., 2022	Sofia, Veliko Tarnovo and Devnya	2016–2020	School	NR	1041	6–15	Non–Cycloplegic	25.55 (22.93–28.32)	25.55 (22.99–28.29)	–
Denmark	Lundberg et al., 2018	Odense (CHAMPS)	2008–2015	Population	NR	307	14–18	Cycloplegic	17.92 (13.79–22.67)	21.92 (17.32–27.35)	I^2^ = 85.4% t^2^ = 0.0302
	Hansen et al., 2020	Copenhagen (CCC2000)	2000–2017	Population	NR	1443	16–17	Non–Cycloplegic	24.95 (22.73–27.26)		
Finland	Aine, 1984	Karkku	NR	Population	89.7	611	6–85	Cycloplegic	11.95 (9.48–14.79)	11.95 (9.61–14.77)	–
Germany	Wolfram et al., 2014; Mirshahi et al., 2016	Frankfurt Rhine–Main (GHS)	2007–2013	Population	60.0	17,411	35–79	Non–Cycloplegic	36.15 (35.44–36.87)	36.15 (35.44–36.87)	–
Hungary	Németh et al., 2022	(CHSP)	2014–2019	NR	NR	69,227	18–99	Non–Cycloplegic	43.24 (42.87–43.61)	43.24 (42.87–43.61)	–
Kazakhstan	Mukazhanova et al., 2022	Almaty	2019	School	93.3	2293	6–16	Cycloplegic	28.26 (26.42–30.15)	28.26 (26.45–30.14)	–
Norway	Midelfar et al., 2002	Nord–Trøndelag (HUNT)	1996–1997	Population	59.9	3137	20–45	Non–Cycloplegic	32.64 (31.00–34.32)	21.73 (11.16–38.03)	I^2^ = 98.4% t^2^ = 0.3168
	Hagen et al., 2018	NR	2015–2016	School	45.6	439	16–19	Non–Cycloplegic	13.44 (10.39–16.99)		
Poland	Czepita et al., 2007; Czepita et al., 2019	Szczecin	2000–2009	NR	95.8	4422	6–18	Cycloplegic	13.30 (12.31–14.33)	13.30 (12.33–14.33)	–
Republic of Ireland	O'Donoghue et al., 2010; French et al., 2012; McCullough et al., 2016	(NICER)	2006–2008; 2012–2014	Population	48.0	1279	6–20	Cycloplegic	13.29 (11.48–15.28)	13.84 (12.63–15.14)	I^2^ = 0% t^2^ = 0
	Harrington et al., 2019; Harrington et al., 2019	(IES)	2016–2018	School	83.3	1626	6–13	Cycloplegic	14.27 (12.60–16.06)		
Russia	Markova et al., 2021	Moscow	2021	NR	NR	523	6–8	NR	20.08 (16.73–23.77)	31.86 (16.61–52.32)	–
	Bikbov et al., 2024	Ufa (UCES)	2019–2022	School	96.0	4737	6–18	Cycloplegic	46.17 (44.74–47.60)		
Sweden	Villarreal et al., 2000	Gothenburg	NR	School	67.0	1045	12–13	Cycloplegic	49.67 (46.59–52.74)	49.67 (46.64–52.69)	–
The Netherlands	Tideman et al., 2018^a^; Tideman et al., 2016; Tideman et al., 2019	Rotterdam (Generation R)	2002–2006	Population	64.2	8175	6–9	Cycloplegic	5.30 (4.82–5.80)	16.40 (6.11–37.16)	I^2^ = 99.9% t^2^ = 0.9402
	Tideman et al., 2018^b^	Rotterdam (RS-III)	2006–2008; 2011–2012	Population and School	NR	2957	≥45	Non–Cycloplegic	36.96 (35.22–38.73)		
	Enthoven et al., 2021	Rotterdam (MAS, Generation R)	2018–2019	Population and School	50.0	238	12–16	Cycloplegic	18.91 (14.14–24.47)		
United Kingdom	Logan et al., 2011	Birmingham (AES)	2006	School	94.6	596	6–13	Cycloplegic	19.46 (16.36–22.87)	30.42 (19.22–44.54)	I^2^ = 99.3% t^2^ = 0.2843
	Sherwin et al., 2012	Norfolk (EPIC)	2006–2010	Population	85.0	4428	48–89	Non–Cycloplegic	27.78 (26.46–29.12)		
	Cumberland et al., 2018	(NCDS)	1958	Population	NR	1985	44–45	Non–Cycloplegic	47.00 (44.79–49.23)		

Studies are ordered alphabetically by country, then by publication status and then by year of survey. ^a^See reference.^35^^b^See reference.^38^ Abbreviations: AES (Aston Eye Study); CCC2000 (Copenhagen Child Cohort 2000 Eye Study); GHS (Gutenberg Health Study); CHAMPS (Childhood Health, Activity, and Motor Performance School Eye Study); CHSP (Comprehensive Health Screening Program of Hungary); EPIC (European Prospective Investigation of Cancer); HUNT (Helseundersøkelse i Nord-Trøndelag Health Study); IES (Ireland Eye Study); MAS (Myopia App Study); NCDS (1958 birth cohort or the National Child Development Study); NICER (Northern Ireland Childhood Errors of Refraction study); NR (not reported); RS-III (Rotterdam Study III); UCES (Ural Children Myopia Study).

All the included studies reported a myopia prevalence of ≤−0.50 D. Only two studies provided data on high myopia, and this was reported using a cut-off value of ≤−5.00 D. Most studies were cross-sectional (n = 17, 77.3%), population-based (n = 11, 50.0%), and included only children (n = 15, 68.2%). Additionally, most studies employed cycloplegia to measure refractive error (n = 12, 54.5%). Of the 22 included studies, five were conducted exclusively in urban areas, one in a rural setting, and six studies included populations from both urban and rural areas (mixed). The remaining ten studies did not specify the recruitment location. In the studies where cycloplegic measures were employed, seven studies used cyclopentolate 1%, one study used tropicamide 0.5%, two studies used 1% tropicamide, one study used 0.8% tropicamide and one study did not report the cycloplegic agent used.

The global prevalence of myopia in Europe was 23.5% (95% CI: 18.5–29.3%), with substantial heterogeneity across the studies (I^2^ = 99.7%). The test for subgroup differences was statistically significant (p < 0.0001), suggesting that the country of origin may influence the reported prevalence of myopia (Table 1). The prevalence of high myopia (≤−5.00 D) was reported by two studies conducted in Sweden (children aged 12–13 years) and the Republic of Ireland (children aged 6–13 years). The pooled prevalence of high myopia was 0.8% (95% CI: 0.2–4.1%), with substantial heterogeneity (I^2^ = 94.7%).

**Fig. 2:**
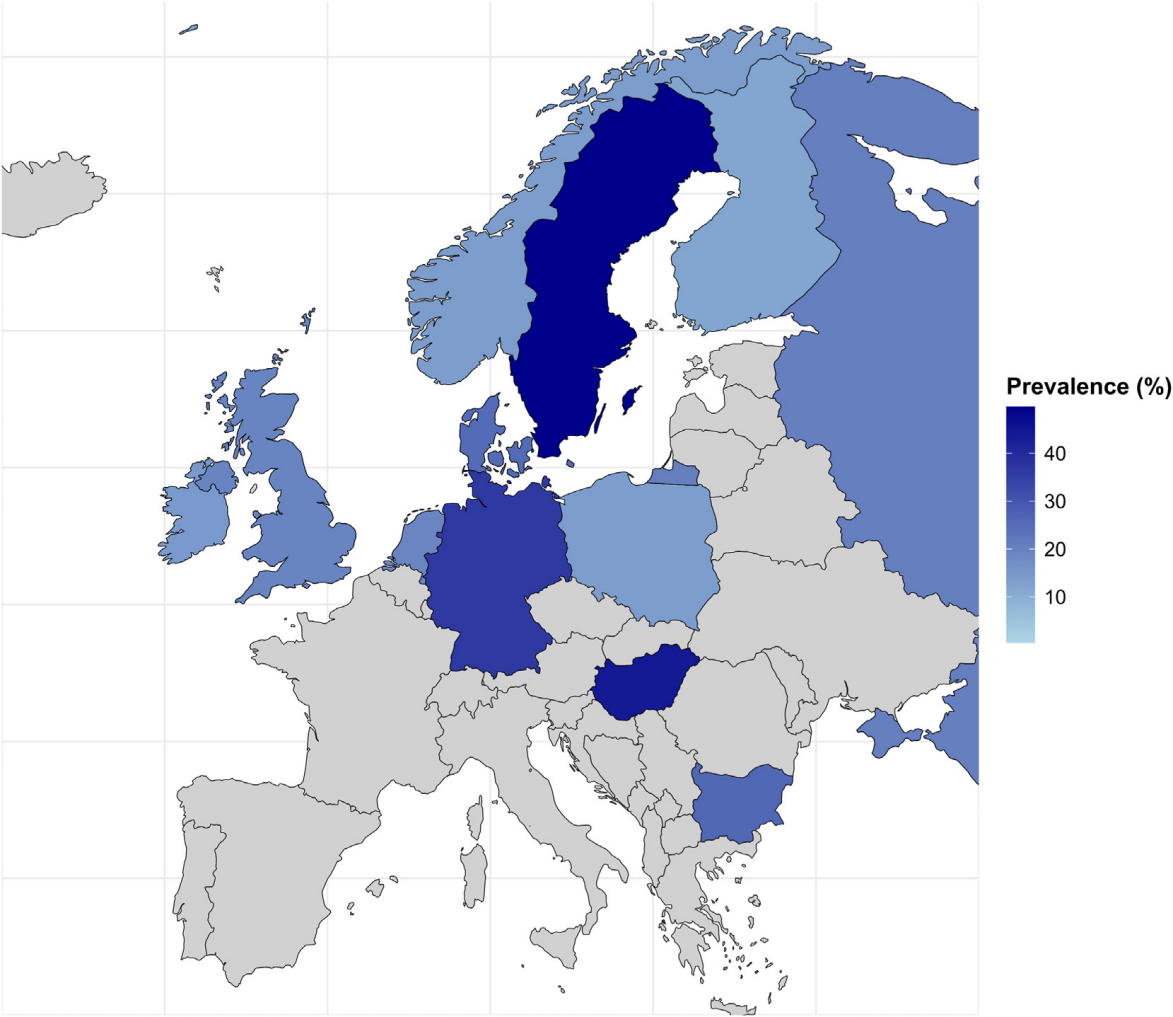
**Pooled prevalence of myopia in Europe by country**. Countries shown in grey were not included in the meta-analysis due to lack of eligible prevalence data. Map boundaries are based on Natural Earth (https://www.naturalearthdata.com) and were generated using the rnaturalearth R package (version 1.0.1). The authors take a neutral position regarding territorial claims and boundary representations.

The country-level analysis revealed significant variation in myopia prevalence across studies (Fig. 2). Finland reported a prevalence of 11.9% (95% CI: 9.6–14.8%), while Sweden had the highest rate at 49.7% (95% CI: 46.6–52.7%). Norway's prevalence was 21.7% (n = 2, 95% CI: 11.2–38.0%), the United Kingdom reported 30.4% (n = 3, 95% CI: 19.2–44.5%), and Bulgaria 25.6% (95% CI: 23.0–28.3%). Lower rates were observed in the Republic of Ireland at 13.8% (n = 2, 95% CI: 12.6–15.1%) and the Netherlands at 16.4% (n = 3, 95% CI: 6.1–37.2%). Higher prevalences were found in Germany (36.2%, n = 1, 95% CI: 35.4–36.9), Russia (31.9%, n = 2, 95% CI: 16.6–52.3%) and Hungary (43.2%, 95% CI: 42.9–43.6%). Heterogeneity within countries was substantial, with I^2^ values exceeding 95% in most cases (and 85% in Denmark), indicating variability in study results. The exception was the Republic of Ireland, where no heterogeneity was observed (I^2^ = 0).

**Fig. 3:**
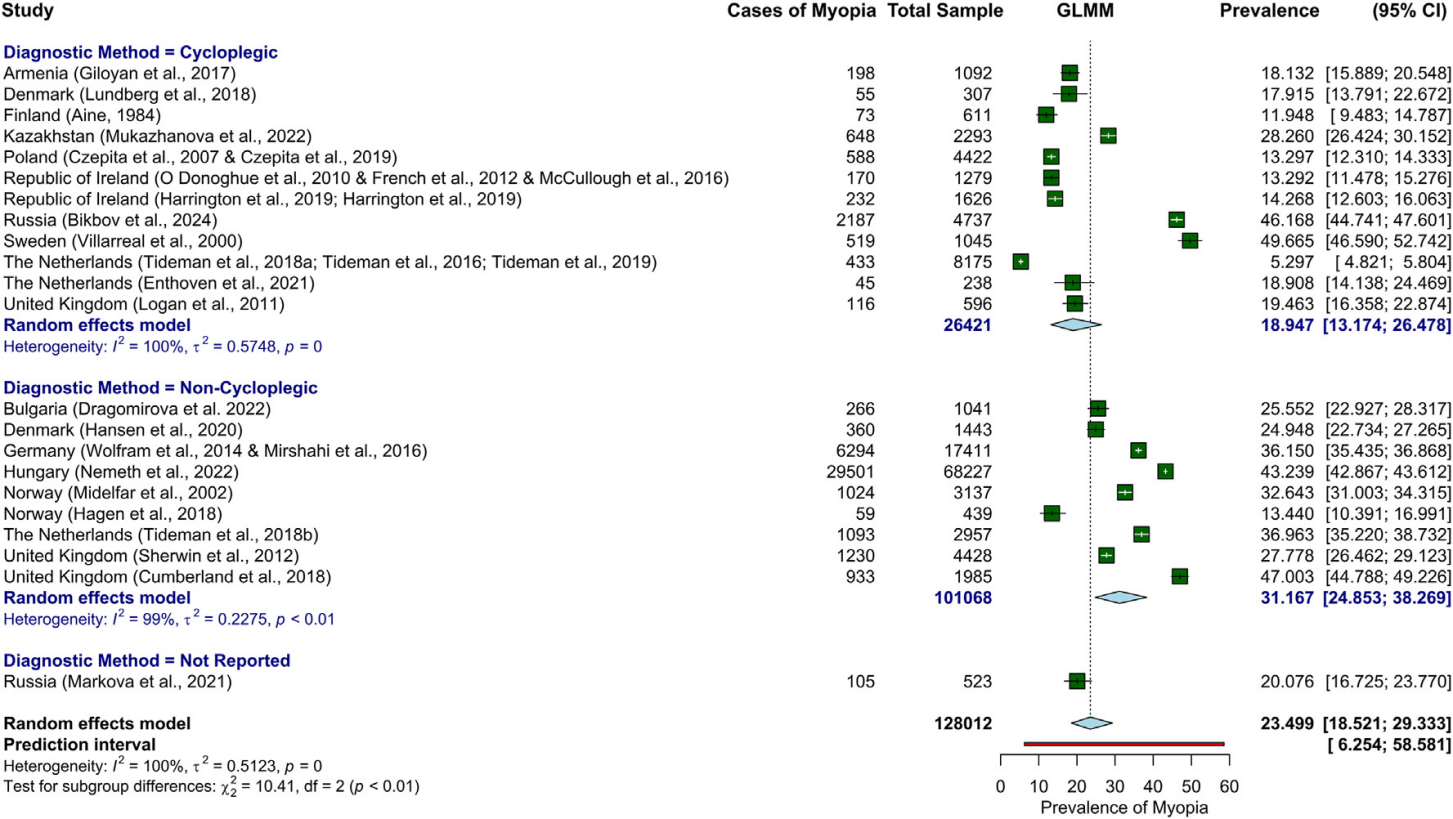
**Pooled prevalence of myopia in Europe based on the diagnostic method (cycloplegic vs. non-cycloplegic refraction)**. GLMM (generalised linear mixed model); CI (confidence interval).

A subgroup analysis was also performed stratified by diagnostic method—cycloplegic vs. non-cycloplegic refraction (Fig. 3). In this subgroup analysis, myopia prevalence varied by diagnostic method. Studies using cycloplegic refraction (n = 12) estimated myopia prevalence at 18.9% (95% CI: 13.2–26.5%; I^2^ = 99.7%), while studies using non-cycloplegic refraction (n = 9) reported a higher prevalence of 31.2% (95% CI: 24.9–38.3%; I^2^ = 99.3%). The single study that did not specify the use of cycloplegia reported a prevalence of 20.1% (95% CI: 16.7–23.8%). The difference between subgroups was statistically significant (p = 0.015 for cycloplegic vs. non-cycloplegic), indicating that diagnostic method may have influenced prevalence estimates. However, substantial heterogeneity was observed within both groups (I^2^ = 99.7% for cycloplegic and 99.3% for non-cycloplegic), suggesting that factors beyond the diagnostic method contribute to variability in prevalence estimates across studies. Univariable meta-regression analyses assessing diagnostic method, country, and recruitment period revealed substantial and persistent residual heterogeneity (I^2^ = 99.7%, 98.2%, and 99.5%, respectively). Studies using non-cycloplegic refraction reported a significantly higher prevalence of myopia compared with those using cycloplegia (odds ratio (OR) = 1.93, 95% CI: 1.11–3.35; p = 0.02). Among the countries included, only Sweden exhibited a statistically significant association with increased myopia prevalence (OR = 4.48, 95% CI: 1.15–17.29; p = 0.03), suggesting that geographical factors may not be a primary determinant of myopia prevalence within this dataset. Myopia prevalence varied significantly by recruitment period, with studies conducted before 2000 reporting higher prevalence than those conducted after 2000 (OR = 2.94, 95% CI: 1.22–7.10; p = 0.02). Among the four studies conducted before 2000, two employed cycloplegic refraction and reported markedly divergent prevalence estimates, 11.9% in Finland and 49.7% in Sweden.

To explore further variable factors influencing myopia prevalence, we performed subgroup analyses including all studies (Table 2) and studies using cycloplegic refraction (Table 3). Significant differences were observed between age groups (p < 0.0001; Supplementary Table S5). Studies that included participants aged ≥18 years reported the highest prevalence of myopia at 32.3% (95% CI: 25.4–40.1%), while studies including children aged 6–17 years reported a lower prevalence of 14.0% (95% CI: 9.0–21.0%; p = 0.0004 between children and adults). The lowest prevalence was found in children aged 6–11 years, with a rate of 6.8% (95% CI: 4.0–11.4%). However, when excluding studies without cycloplegic refraction, the subgroup analysis of myopia prevalence in Europe (Table 3) revealed a different trend. In this subset, while children aged 6–11 years still showed a lower prevalence of myopia (5.5% 95% CI: 3.8–7.8%), children aged 12–17 years had a similar prevalence (25.2%, 95% CI: 18.0–34.1%) to participants aged 18–39 years (24.3%, 95% CI: 17.8–32.2%; p = 0.87 between children aged 12–17 years and adults).

**Table 2:** Summary statistics of subgroup meta-analysis of the global prevalence of myopia in Europe.

	Prevalence (95% CI)	95% prediction interval	Studies (n)	Participants (n)	*I*^*2*^ (95% CI)	*P*_*heterogeneity*_	*P*_*difference*_
By age							**<0.0001**
Children							
6−11 years	6.78 [ 3.97; 11.35]	[0.913; 36.479]	6	12,640	97.3% [95.8%; 98.2%]	<0.0001	
12−17 years	23.00 [16.80; 30.65]	[6.621; 55.735]	8	6540	98.2% [97.5%; 98.7%]	<0.0001	
Adults							
18−39 years	32.74 [21.62; 46.21]	[5.097; 81.521]	5	26,306	99.1% [98.7%; 99.4%]	<0.0001	
+40 years	31.72 [24.20; 40.33]	[9.564; 67.106]	5	11,883	98.7% [98.0%; 99.1%]	<0.0001	
By sex							0.1367
Female	22.96 [16.74; 30.63]	[6.735; 55.142]	7	5754	96.8% [95.2%; 97.9%]	<0.0001	
Male	19.80 [12.76; 29.40]	[3.560; 62.269]	7	5362	97.5% [96.3%; 98.3%]	<0.0001	
By recruitment location							0.4124
Urban	23.52 [14.86; 35.13]	[3.181; 74.212]	5	10,191	99.4% [99.2%; 99.6%]	<0.0001	
Rural & Mixed	18.85 [13.95; 24.97]	[5.887; 46.311]	7	11,987	99.2% [98.9%; 99.4%]	<0.0001	
By region							0.3854
Northern Europe	22.92 [16.66; 30.66]	[5.805; 58.924]	11	16,896	99.0% [98.8%; 99.2%]	<0.0001	
Eastern Europe	23.71 [15.57; 34.39]	[3.786; 71.057]	5	13,067	99.7% [99.6%; 99.7%]	<0.0001	
Western Europe	20.40 [ 9.09; 39.64]	[0.256; 96.244]	4	28,781	99.9% [99.8%; 99.9%]	<0.0001	
Southeast & Central Europe	33.96 [22.82; 47.21]	[16.545; 57.151]	2	69,268	99.1% [98.8%; 99.3%]	<0.0001	
By period of recruitment							0.1160
Before 2000	32.98 [18.58; 51.49]	[1.134; 95.478]	4	6778	99.0% [98.6%; 99.4%]	<0.0001	
2000–2010	14.36 [7.68; 25.26]	[0.539; 83.831]	4	17,621	99.7% [99.6%; 99.8%]	<0.0001	
2011–2022	24.01 [17.73; 31.66]	[6.941; 57.237]	9	80,216	99.4% [99.2%; 99.5%]	<0.0001	
By site							0.6915
Population based	22.68 [15.17; 32.49]	[4.106; 66.765]	10	41,733	99.7% [99.6%; 99.7%]	<0.0001	
School based	25.15 [17.57; 34.64]	[5.812; 64.671]	8	12,869	99.3% [99.1%; 99.4%]	<0.0001	

Abbreviations: n (number); CI (confidence interval). Values in bold indicate statistically significant differences in myopia prevalence between subgroups (p  <  0.05), based on random-effects models.

**Table 3:** Summary statistics of subgroup meta-analysis of myopia prevalence in Europe, based on studies that employed cycloplegic refraction.

	Prevalence (95% CI)	95% prediction interval	Studies (n)	Participants (n)	*I*^*2*^ (95% CI)	*P*_*heterogeneity*_	*P*_*difference*_
By age							**< 0.0001**
Children							
6−11 Years	5.45 [3.76; 7.82]	[1.349; 19.518]	5	12,117	90.3% [80.1%; 95.2%]	<0.0001	
12−17 Years	25.19 [17.96; 34.13]	[6.439; 62.236]	6	4851	98.6% [98.0%; 99.0%]	<0.0001	
Adults							
18−39 Years	24.29 [17.84; 32.15]	[0.387; 96.365]	3	497	81.6% [43.0%; 94.1%]	<0.0001	
+40 Years	–	–	–	–	–	–	
By sex							0.7394
Female	18.85 [13.66; 25.42]	[3.910; 56.992]	4	1843	93.0% [85.3%; 96.7%]	<0.0001	
Male	17.27 [11.25; 25.57]	[1.994; 68.161]	4	1806	93.7% [87.0%; 96.9%]	<0.0001	
By recruitment location							0.0822
Urban	27.20 [14.42; 45.32]	[0.001; 99.991]	3	8309	99.6% [99.4%; 99.7%]	<0.0001	
Rural & Mixed	14.87 [12.87; 17.12]	[8.856; 23.898]	5	7989	82.9% [61.1%; 92.5%]	<0.0001	
By region							0.1989
Northen Europe	19.21 [12.16; 28.99]	[3.099; 63.858]	6	5464	99.1% [98.8%; 99.3%]	<0.0001	
Eastern Europe	24.67 [14.74; 38.29]	[1.414; 88.205]	4	11,544	99.7% [99.7%; 99.8%]	<0.0001	
Western Europe	10.04 [4.00; 23.01]	[2.023; 37.647]	2	8413	98.5% [96.8%; 99.3%]	<0.0001	
By period of recruitment							0.0980
Before 2000	26.85 [8.46; 59.30]	[3.284; 79.865]	2	1656	99.5% [99.2%; 99.7%]	<0.0001	
2000–2010	11.25 [5.97; 20.18]	[0.002; 99.893]	3	13,193	99.3% [99.0%; 99.6%]	<0.0001	
2011–2022	23.79 [15.72; 34.33]	[3.944; 70.366]	5	9986	99.5% [99.3%; 99.6%]	<0.0001	
By site							**0.0029**
Population based	11.05 [7.03; 16.94]	[1.125; 57.538]	4	10,372	98.3% [97.3%; 99.0%]	<0.0001	
School based	27.52 [18.07; 39.54]	[4.761; 74.257]	6	11,389	99.4% [99.2%; 99.5%]	<0.0001	

Abbreviations: n (number); CI (confidence interval). Values in bold indicate statistically significant differences in myopia prevalence between subgroups (p < 0.05), based on random-effects models.

In the subgroup analysis by recruitment location (Table 2, Table 3), myopia prevalence did not differ significantly between urban and “rural & mixed” clusters (p = 0.41 for all studies and p = 0.082 for studies using cycloplegic refraction). Urban studies tended to report higher prevalence rates, with an overall prevalence of 23.5% (95% CI: 14.9–35.1%) and 27.2% (95% CI: 14.4–45.3%) in studies employing cycloplegic refraction. The single rural study using cycloplegic refraction reported the lowest prevalence of 11.9% (95% CI: 9.6–14.8%). The “rural & mixed” cluster presented intermediate prevalences, 18.9% (95% CI: 14.0–25.0%) overall and 14.9% (95% CI: 12.9–17.1%) in cycloplegic refraction studies. Mixed setting studies alone showed similar results, with overall prevalences of 20.2% (95% CI: 14.9–26.9%) and 15.5% (95% CI: 13.4–18.0%) for cycloplegic refraction.

Further subgroup analysis (Table 2, Table 3) showed that the prevalence of myopia did not differ by sex and region. In contrast, a higher prevalence of myopia was found in school-based studies compared to population-based studies. Furthermore, our meta-analysis of myopia prevalence did not show significant differences between recruitment periods (before 2000 vs. 2000–2010 vs. 2011–2022; p = 0.12 for all studies and p = 0.098 for studies using cycloplegic refraction). However, when the analysis was restricted to more recent studies (excluding those before 2000), a significant increase in myopia prevalence in Europe was observed between 2000–2010 and 2011–2022 for studies using cycloplegia (p = 0.040).

**Fig. 4:**
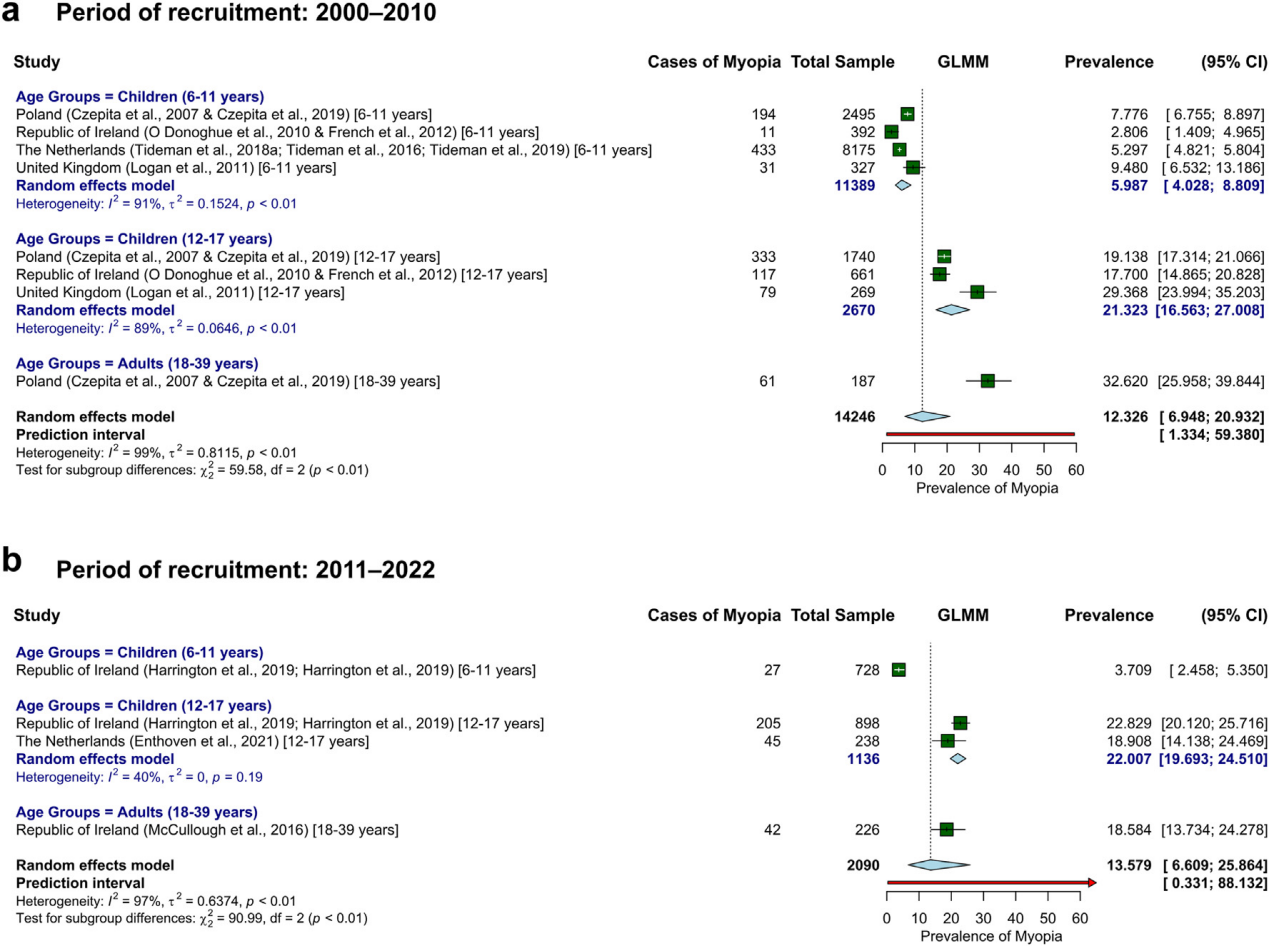
**Pooled prevalence of myopia in Europe by age groups (6–11, 12–17, and 18–39 years), stratified by period of recruitment (a) 2000–2010 and (b) 2011–2022, based on studies using cycloplegia**.

An analysis of pooled data, stratified by age group and recruitment period (2000–2010 vs. 2011–2022; Fig. 4) did not reveal a significant temporal trend in myopia prevalence. These results should be interpreted with caution given the limited sample size and the exploratory nature of the subgroup analyses. With similar caution, we note that among adolescents (12–17 years), prevalence remained comparable between the two periods (21.3% for 2000–2010 and 22.0% for 2011–2022), with lower heterogeneity (I^2^ = 88.5% and I^2^ = 40.5%, respectively). A similar pattern was observed in children aged 6–11 years, with prevalence remaining relatively stable (6.0% for 2000–2010 and 3.7% for 2011–2022).

An additional exploratory analysis was also performed on pooled myopia prevalence from studies using cycloplegia, stratified by age group and study site (Supplementary Figure S2). This analysis revealed no substantial discrepancy in myopia prevalence between school-based and population-based studies. In children aged 6–11 years, prevalence was 5.9% in school-based studies and 5.2% in population-based studies. In adolescents aged 12–17 years, prevalence was 25.2% and 17.7%, respectively.

The initial funnel plot analysis indicated asymmetry, which was further validated by both Egger's linear regression test and Peters' regression test, yielding statistically significant results (p = 0.018 and p = 0.047, respectively; see Supplementary Figures S3 and S4). However, when the analysis was restricted to studies employing cycloplegic refraction, no asymmetry was observed, and both tests showed no significant asymmetry (p = 0.51 and p = 0.90, respectively; Supplementary Figures S5 and S6). This suggests that the observed asymmetry is primarily due to the inclusion of studies not using cycloplegic refraction, rather than publication bias alone. To further explore potential biases related to study size, a meta-regression analysis was conducted with study size as a predictor variable. The results showed no significant relationship between study size and myopia prevalence (OR = 1.19, 95% CI: 0.96–1.48; p = 0.11), suggesting that study size did not significantly influence the prevalence estimates. This supports the interpretation that small study effects were not a substantial source of bias in the analysis. Consequently, the observed variability in prevalence estimates is more likely to be influenced by other factors, such as the use of cycloplegia in the diagnostic assessment and differences in age groups.

## Discussion

We compiled data on the prevalence of myopia from 14 countries in Europe, including 128,012 participants, and estimated an overall prevalence of 23.5% (95% CI: 18.5–29.3; I^2^ = 99.7%), which is comparable to previous estimates (22.6%).^47^^,^^48^ Importantly, when the most reliable diagnostic methods (cycloplegia) are taken into consideration, the prevalence appears to be lower than initially anticipated (18.9%, 95% CI: 13.2–26.5%; I^2^ = 99.7%).

We observed a wide variability in the estimate across countries in Europe, ranging from 11.9% in Finland to 49.7% in Sweden. Moreover, myopia prevalence was generally higher in the 12–17 years age group than in children aged 6–11 years. Our comparison of population-based and school-based studies revealed a higher prevalence of myopia in school-based studies. Notably, this difference was mainly due to the inclusion of children aged 6–11 years in several population-based studies, a group typically associated with a lower myopia prevalence and not related with education settings. Indeed, when the data were further analysed by age group and study site, this difference was clarified. The prevalence of myopia did not show a significant difference between school-based and population-based studies when stratified by age. This suggests that while age distribution influenced the results, the type of study site alone did not fully explain the observed differences. Moreover, when adjusting for methodological quality (only studies that used cycloplegic refraction), the prevalence of myopia in adolescents aged 12–17 years remained higher than in children aged 6–11 years, but comparable to that in individuals aged 18–39 years. Additionally, among the subset of studies that reported sex-specific data, we found no significant differences in myopia prevalence between sexes. This finding suggests that the observed variability in myopia prevalence across Europe is primarily attributable to differences in age and diagnostic methods, rather than sex. However, this interpretation is constrained by the limited availability of sex-disaggregated data.

The European prevalence figures are notably lower than in East Asia, where ^17^^,^^47^ higher rates have been attributed to intense academic pressure and early education.^48^ Nevertheless, there is a debate on whether those estimates are region depend as it seems that East Asians are the most affected by the burden of myopia.^49^ According to Holden et al. (2016), the European prevalence of myopia is anticipated to rise to 50% by 2050, driven by lifestyle shifts, particularly increased near work and decreased outdoor exposure.^17^ Our results show an increase in myopia prevalence in the last decade (2011–2022) compared to 2000–2010 when the meta-analysis was restricted to studies using cycloplegic refraction, suggesting a higher prevalence of myopia over time in Europe. However, when the data were stratified by age group and recruitment period, no increase in the prevalence of myopia was observed. These findings suggest that the rise in prevalence in Europe over time may not be as pronounced as previously anticipated. Given the limited number of studies included and the exploratory nature of the subgroup analyses, which may not have accounted for all potential confounders and biases, these results should be interpreted with caution. Further research with larger and more representative samples is needed to draw more definitive conclusions.

In a previous meta-analysis of population-based studies published in 2015 the authors reported increases in myopia prevalence in Western and Northern Europe.^11^ However, our meta-analysis considering cycloplegic studies, shows that there was a non-significant trend for a higher prevalence of myopia in Eastern Europe compared to other regions. In some European countries myopia has been reported to remain stable, especially in Nordic countries such as Denmark.^50^ Particularly, conflicting data from Sweden has been published.^19^^,^^51^^,^^52^ Due to the design and methods used in some of these Swedish studies, such as retrospective design and non-randomised samples, they were excluded from our meta-analysis.

In our study, the prevalence of myopia was found to differ significantly based on whether cycloplegic or non-cycloplegic refraction was used. Studies using cycloplegic refraction reported a myopia prevalence of 18.9%, while non-cycloplegic studies estimated a significantly higher prevalence of 31.2%. This difference aligns with previous studies, which show that non-cycloplegic refraction overestimates myopia prevalence due to accommodation, particularly in younger populations.^53^, ^54^, ^55^, ^56^ In addition, we found no significant evidence of publication bias when only studies using cycloplegic refraction were considered. This suggests that the observed differences in prevalence estimates are likely due to methodological factors, such as the use of cycloplegia in the diagnostic assessment and variations in age groups, rather than publication bias or small study effects. The consistent myopic shift in non-cycloplegic refraction highlights the need for standardised cycloplegic protocols in epidemiological studies to improve accuracy in the reporting of prevalence rates. Cycloplegic methods are essential for reliable data, especially in settings where precise prevalence measurements are critical to understand and address myopia prevalence and progression. Moreover, in the quality analysis, we found that studies with one or more negative items on the quality assessment checklist, conducted in Sweden, Norway, the United Kingdom, Germany, the Netherlands (Rotterdam, RS-III), Hungary and Russia reported myopia prevalence greater than 30%.^19^^,^^20^^,^^26^, ^27^, ^28^^,^^38^^,^^44^^,^^46^ These findings highlight significant areas for improvement in study design and reporting to generate evidence on myopia prevalence.

Previous research has shown that children in urban settings have higher odds of developing myopia compared to those in rural settings.^49^ In the present study, although the prevalence of myopia exhibited a tendency to be higher in urban areas, these differences were not statistically significant when compared to rural or mixed settings. These results may be attributed to the ongoing urbanisation process, which may lead to education, lifestyle, and social environments in rural areas becoming more comparable to those in urban settings.^57^

To reduce the burden of myopia, countries need to adopt comprehensive national action plans that promote high-quality research on the epidemiology of myopia and develop effective prevention, early detection and management programmes. In addition, it is critical to prioritise myopia detection and management as a standard of care, while building the capacity of primary eye care professionals and promoting interdisciplinary collaboration.

Preventing high myopia and pathological myopia should remain a global key strategy. This can be achieved through extended health promotion efforts, including educational and comprehensive lifestyle programmes that encourage increased outdoor time to reduce myopia complications.^58^ The World Health Organization (WHO) has developed a toolkit to assist policy and decision makers as well as implementers to establish large-scale educational programmes for key populations groups.^59^ Such programmes should be implemented in Europe to reduce the incidence of myopia and avoid the development of visual impairment. Further research on the cost-effectiveness of systematic screening for myopia should be undertaken, especially in countries where the prevalence is higher, if resources are available.

Our study should be interpreted considering several limitations. First, we included 22 studies, of which 10 (45.5%) did not use cycloplegia to measure refractive error, potentially leading to an overestimation of myopia prevalence. Nonetheless, in our pooled subgroup analysis, which included studies with a lower risk of bias, the overall estimate was considerably reduced to 18.9% (95% CI: 13.2–26.5%), although some subgroups had a limited number of studies. Consistent with other prevalence meta-analyses, we observed substantial heterogeneity between studies. Univariable meta-regression analyses assessing diagnostic method, country, and recruitment period, showed that heterogeneity remained substantial. However, when myopia prevalence was pooled by age group and stratified by cycloplegic assessment, a reduction in heterogeneity was observed, suggesting that age may contribute to the variation in prevalence. These results, however, arise from exploratory subgroup analyses and should be interpreted with caution due to the increased risk of false-positive findings and the lack of adjustment for potential confounders. These findings highlight the importance of future studies employing cycloplegic refraction and reporting prevalence using individual-level data, or at least stratifying by age, to validate observed patterns. This approach would enhance comparability and provide a clearer understanding of myopia prevalence across different populations. In addition, there was a discrepancy in country representation, with some countries having only a single small-scale study, while others had no data available. This may affect the generalisability of the findings. Future research would benefit from exploring the impact of risk factors such as time spent outdoors, near work activities, and parental myopia, information that was absent in most studies included in the review, limiting the ability to fully explore the role of these well-established risk factors. This lack of data impaired the assessment of the influence of lifestyle and genetic predispositions in the myopia prevalence.

Despite these limitations, to the best of our knowledge, this study is the first systematic review and meta-analysis to provide a comprehensive summary of existing knowledge on the prevalence of myopia in Europe, by diagnostic method. We published a protocol of our methods before we undertook the study, and we used rigorous methodological and statistical procedures to obtain and pool data from about 128,012 participants. Furthermore, subgroup analyses and meta-regression analyses were done to investigate the various factors that may influence our estimates. We applied strict criteria for study selection to ensure high methodological quality and representativeness of the findings. As a result, studies deemed unrepresentative were excluded, resulting in the omission of data from several European countries (e.g., Portugal, Spain, among others). While this approach may have enhanced the reliability of our results, it limits the geographical scope and could reduce the generalisability of the findings across the entire region. Thus, the prevalence estimates presented in our study may not fully capture the diversity of prevalence across Europe, particularly in regions where studies were either unavailable or excluded in accordance with our predefined eligibility criteria.

In conclusion, this study provides a foundation to understand the prevalence of myopia in Europe and highlights the urgent need for standardised methodologies, particularly the use of cycloplegic refraction, as well as detailed age-specific data. Such data are essential for accurately assessing myopia prevalence across different populations. These high-quality data are crucial for effective public health monitoring and intervention, helping to address the myopia epidemic and mitigate its associated social and economic burden.

## Contributors

AM-R, AG, and CL conceived the study and developed the protocol. AM-R and CL did the literature search, selected the studies, extracted the relevant information and synthesised the data. AM-R and CL wrote the first draft of the paper. AG critically revised successive drafts of the paper and all authors approved its final version. All authors had full access to all the data in the study and had final responsibility for the decision to submit for publication.

## Data sharing statement

All data generated or analysed during this study were included in the main article and its supplementary information files. The study protocol is publicly available in the PROSPERO database (CRD42023471527). Additional details or clarifications related to this meta-analysis are available from the corresponding authors upon reasonable request.

## Editor note

The Lancet Group takes a neutral position with respect to territorial claims in published maps and institutional affiliations.

## Declaration of interests

Carla Lanca reports a relationship with Eyerising that includes consulting or advisory. Andrzej Grzybowski reports a relationship with Alcon, Bausch&Lomb, Zeiss, Teleon, J&J, CooperVision, Hoya, Essilor, Thea, Santen, Polpharma, Viatris & Alcon that includes funding grants. Andrzej Grzybowski reports a relationship with Thea, Santen, Polpharma, Viatris, Eyerising, Essilor & Alcon that includes speaking and lecture fees. Nevakar, Santen, GoCheckKids & Thea reports a relationship with Andrzej Grzybowski that includes board membership. André Moreira-Rosário reports having no competing interests.

## Appendix A Supplementary data

Supplementary Material, Figures and Tables

Summary in Portuguese
