# Supplementary material for: Prevalence of myopia in Europe: a systematic review and meta-analysis of data from 14 countries
Source: Lancet Reg Health Eur. 2025 May 22;54:101319. doi: 10.1016/j.lanepe.2025.101319 (PMC12266183; doi:10.1016/j.lanepe.2025.101319)
Supplement: Supplementary Material, Figures and Tables [file mmc1.docx]

Supplementary Data of

Prevalence of myopia in Europe: a systematic review and meta-analysis of data from 14 countries

Table of Contents

[**Supplementary Table 1**: Query definition. **2**](#_Toc191912255)

[**Supplementary Table 2**: Query results. **3**](#_Toc191912256)

[**Supplementary Material 1**: Data selection, extraction, management, and analysis procedures. **4**](#_Toc191912257)

[**Supplementary Table 3**: PRISMA 2020 Checklist. **7**](#_Toc191912258)

[**Supplementary Table 4**: MOOSE Checklist. **14**](#_Toc191912259)

[**Supplementary Figure 1**: Summary of the quality assessment. **18**](#_Toc191912260)

[**Supplementary Table 5**: Prevalence of myopia by study and age group. **19**](#_Toc191912261)

[**Supplementary Figure 2**: Pooled prevalence of myopia in Europe by age groups stratified by study site.](#_Toc191912262) [**21**](#_Toc191912263)

[**Supplementary Figure 3**: Funnel plot using the Freeman-Tukey double arcsine transformation (FTDAT). **22**](#_Toc191912264)

[**Supplementary Figure 4**: Funnel plot using the generalised linear mixed model (GLMM). **23**](#_Toc191912265)

[**Supplementary Figure 5**: Funnel plot of studies employing cycloplegic refraction, using the FTDAT. **24**](#_Toc191912266)

[**Supplementary Figure 6**: Funnel plot of studies employing cycloplegic refraction, using the GLMM. **25**](#_Toc191912267)

**Supplementary Table 1:** Query definition with “Myopia” combined with “Prevalence” and “Europe” (#1 AND #2 AND #3).

| **#** | **PUBMED** | **SCOPUS** | **WEB OF SCIENCE** |
| --- | --- | --- | --- |
| **1** | ("Myopia"[MeSH Terms] OR "Myopia"[Title/Abstract] OR "short sight*" [Title/Abstract] OR "Refractive Errors"[MeSH Terms] OR "Refractive Errors"[Title/Abstract] OR "ocular refraction"[Title/Abstract] OR "refraction, ocular"[MeSH Terms]) | TITLE-ABS-KEY ("Myopia" ) OR TITLE-ABS-KEY ( "short sight*" ) OR TITLE-ABS-KEY ( "Refractive Errors" ) OR TITLE-ABS-KEY ( "ocular refraction" ) | TS=("Myopia" OR "short sight*" OR "Refractive Errors" OR "ocular refraction" OR "refraction, ocular") |
| **2** | ("Prevalence"[MeSH Terms] OR "Prevalence"[Title/Abstract] OR "prevalen*"[Title/Abstract]) | TITLE-ABS-KEY ( "Prevalence") OR TITLE-ABS-KEY ( "prevalen*" ) | TS=("Prevalence" OR "prevalen*") |
| **3** | **("Europe"[MeSH Terms] OR "European Union"[MeSH Terms] OR "europ*"[Title/Abstract] OR "Albania"[MeSH Terms] OR "Albania"[Title/Abstract] OR "Albanian"[Title/Abstract] OR "Andorra"[MeSH Terms] OR "Andorra"[Title/Abstract] OR "Andorran"[Title/Abstract] OR "Austria"[MeSH Terms] OR "Austria"[Title/Abstract] OR "Austrian"[Title/Abstract] OR "Belarus"[Title/Abstract] OR "Belarusian"[Title/Abstract] OR "Belgium"[MeSH Terms] OR "Belgium"[Title/Abstract] OR "Belgian"[Title/Abstract] OR "Bosnia and Herzegovina"[MeSH Terms] OR "Bosnia and Herzegovina"[Title/Abstract] OR "Bosnian"[Title/Abstract] OR "Bulgaria"[MeSH Terms] OR "Bulgaria"[Title/Abstract] OR "Bulgarian"[Title/Abstract] OR "Croatia"[MeSH Terms] OR "Croatia"[Title/Abstract] OR "Croatian"[Title/Abstract] OR "Cyprus"[MeSH Terms] OR "Cyprus"[Title/Abstract] OR "Cypriot"[Title/Abstract] OR "Czech Republic"[MeSH Terms] OR "Czech Republic"[Title/Abstract] OR "Czech"[Title/Abstract] OR "Denmark"[MeSH Terms] OR "Denmark"[Title/Abstract] OR "Danish"[Title/Abstract] OR "Estonia"[MeSH Terms] OR "Estonia"[Title/Abstract] OR "Estonian"[Title/Abstract] OR "Finland"[MeSH Terms] OR "Finland"[Title/Abstract] OR "Finnish"[Title/Abstract] OR "France"[MeSH Terms] OR "France"[Title/Abstract] OR "French"[Title/Abstract] OR “Germany"[MeSH Terms] OR "Germany"[Title/Abstract] OR "German"[Title/Abstract] OR "Greece"[MeSH Terms] OR "Greece"[Title/Abstract] OR "Greek"[Title/Abstract] OR "Hungary"[MeSH Terms] OR "Hungary"[Title/Abstract] OR "Hungarian"[Title/Abstract] OR "Iceland"[MeSH Terms] OR "Iceland"[Title/Abstract] OR "Icelandic"[Title/Abstract] OR "Ireland"[MeSH Terms] OR "Ireland"[Title/Abstract] OR "Irish"[Title/Abstract] OR "Italy"[MeSH Terms] OR "Italy"[Title/Abstract] OR "Italian"[Title/Abstract] OR "Kazakhstan"[MeSH Terms] OR "Kazakhstan"[Title/Abstract] OR "Kazakhstani"[Title/Abstract] OR "Kosovo"[MeSH Terms] OR "Kosovo"[Title/Abstract] OR "Kosovan"[Title/Abstract] OR "Latvia"[MeSH Terms] OR "Latvia"[Title/Abstract] OR "Latvian"[Title/Abstract] OR "Liechtenstein"[MeSH Terms] OR "Liechtenstein"[Title/Abstract] OR "Lithuania"[MeSH Terms] OR "Lithuania"[Title/Abstract] OR "Lithuanian"[Title/Abstract] OR "Luxembourg"[MeSH Terms] OR "Luxembourg"[Title/Abstract] OR "Luxembourgish"[Title/Abstract] OR "Malta"[MeSH Terms] OR "Malta"[Title/Abstract] OR "Maltese"[Title/Abstract] OR "Moldova"[MeSH Terms] OR "Moldova"[Title/Abstract] OR "Moldovan"[Title/Abstract] OR "Monaco"[MeSH Terms] OR "Monaco"[Title/Abstract] OR "Monegasque"[Title/Abstract] OR "Montenegro"[MeSH Terms] OR "Montenegro"[Title/Abstract] OR "Montenegrin"[Title/Abstract] OR "Netherlands"[MeSH Terms] OR "Netherlands"[Title/Abstract] OR "Dutch"[Title/Abstract] OR "North Macedonia"[Title/Abstract] OR "North Macedonian"[Title/Abstract] OR "Norway"[MeSH Terms] OR "Norway"[Title/Abstract] OR "Norwegian"[Title/Abstract] OR "Poland"[MeSH Terms] OR "Poland"[Title/Abstract] OR "Polish"[Title/Abstract] OR "Portugal"[MeSH Terms] OR "Portugal"[Title/Abstract] OR "Portuguese"[Title/Abstract] OR "Romania"[MeSH Terms] OR "Romania"[Title/Abstract] OR "Romanian"[Title/Abstract] OR "Russia"[MeSH Terms] OR "Russia"[Title/Abstract] OR "Russian"[Title/Abstract] OR "San Marino"[MeSH Terms] OR "San Marino"[Title/Abstract] OR "Serbia"[MeSH Terms] OR "Serbia"[Title/Abstract] OR "Serbian"[Title/Abstract] OR "Slovakia"[MeSH Terms] OR "Slovakia"[Title/Abstract] OR "Slovak"[Title/Abstract] OR "Slovenia"[MeSH Terms] OR "Slovenia"[All Fields] OR "Slovenia's"[All Fields] OR "Spain"[Mesh] OR "Spain"[Title/Abstract] OR "Spanish"[Title/Abstract] OR "Sweden"[MeSH Terms] OR "Sweden"[Title/Abstract] OR "Swedish"[Title/Abstract] OR "Switzerland"[MeSH Terms] OR "Switzerland"[Title/Abstract] OR "Turkey"[MeSH Terms] OR "Turkey"[Title/Abstract] OR "Turkish"[Title/Abstract] OR "United Kingdom"[MeSH Terms] OR "United Kingdom"[Title/Abstract] OR "England"[Title/Abstract] OR "Wales"[Title/Abstract] OR "Scotland"[Title/Abstract] OR "Northern Ireland"[Title/Abstract] OR "Vatican City"[MeSH Terms] OR "Vatican City"[Title/Abstract])** | **TITLE-ABS-KEY ( "Europe" OR "European Union" OR "europ*" OR "Albania" OR "Albanian" OR "Andorra" OR "Andorran" OR "Austria" OR "Austrian" OR "Belarus" OR "Belarusian" OR "Belgium" OR "Belgian" OR "Bosnia and Herzegovina" OR "Bosnian" OR "Bulgaria" OR Bulgarian" OR "Croatia" OR "Croatian" OR "Cyprus" OR "Cypriot" OR "Czech Republic" OR "Czech" OR "Denmark" OR "Danish" OR "Estonia" OR "Estonian" OR "Finland" OR "Finnish" OR "France" OR "French" OR "Germany" OR "German" OR "Greece" OR "Greek" OR "Hungary" OR "Hungarian" OR "Iceland" OR "Icelandic" OR "Ireland" OR "Irish" OR "Italy" OR "Italian" OR "Kazakhstan" OR "Kazakhstani" OR "Kosovo" OR "Kosovan" OR "Latvia" OR "Latvian" OR "Liechtenstein" OR "Lithuania" OR "Lithuanian" OR "Luxembourg" OR "Luxembourgish" OR "Malta" OR "Maltese" OR "Moldova" OR "Moldovan" OR "Monaco" OR "Monegasque" OR "Montenegro" OR "Montenegrin" OR "Netherlands" OR "Dutch" OR "North Macedonia" OR "North Macedonian" OR "Norway" OR "Norwegian" OR "Poland" OR "Polish" OR "Portugal" OR "Portuguese" OR "Romania" OR "Romanian" OR "Russia" OR "Russian" OR "San Marino" OR "Serbia" OR "Serbian" OR "Slovakia" OR "Slovak" OR "Slovenia" OR "Spain" OR "Spanish" OR "Sweden" OR "Swedish" OR "Switzerland" OR "Turkey" OR "Turkish" OR "United Kingdom" OR "England" OR "Wales" OR "Scotland" OR "Northern Ireland" OR "Vatican City" )** | **TS=("Europe" OR "European Union" OR "europ*" OR "Albania" OR "Albanian" OR "Andorra" OR "Andorran" OR "Austria" OR "Austrian" OR "Belarus" OR "Belarusian" OR "Belgium" OR "Belgian" OR "Bosnia and Herzegovina" OR "Bosnian" OR "Bulgaria" OR "Bulgarian" OR "Croatia" OR "Croatian" OR "Cyprus" OR "Cypriot" OR "Czech Republic" OR "Czech" OR "Denmark" OR "Danish" OR "Estonia" OR "Estonian" OR "Finland" OR "Finnish" OR "France" OR "French" OR "Germany" OR "German" OR "Greece" OR "Greek" OR "Hungary" OR "Hungarian" OR "Iceland" OR "Icelandic" OR "Ireland" OR "Irish" OR "Italy" OR "Italian" OR "Kazakhstan" OR "Kazakhstani" OR "Kosovo" OR "Kosovan" OR "Latvia" OR "Latvian" OR "Liechtenstein" OR "Lithuania" OR "Lithuanian" OR "Luxembourg" OR "Luxembourgish" OR "Malta" OR "Maltese" OR "Moldova" OR "Moldovan" OR "Monaco" OR "Monegasque" OR "Montenegro" OR "Montenegrin" OR "Netherlands" OR "Dutch" OR "North Macedonia" OR "North Macedonian" OR "Norway" OR "Norwegian" OR "Poland" OR "Polish" OR "Portugal" OR "Portuguese" OR "Romania" OR "Romanian" OR "Russia" OR "Russian" OR "San Marino" OR "Serbia" OR "Serbian" OR "Slovakia" OR "Slovak" OR "Slovenia" OR "Spain" OR "Spanish" OR "Sweden" OR "Swedish" OR "Switzerland" OR "Turkey" OR "Turkish" OR "United Kingdom" OR "England" OR "Wales" OR "Scotland" OR "Northern Ireland" OR "Vatican City")** |

# Supplementary Table 2: Query results with “Myopia” combined with “Prevalence” and “Europe” (#1 AND #2 AND #3).

| ****#**** | ****PUBMED**** | ****SCOPUS**** | ****WEB OF SCIENCE**** |
| --- | --- | --- | --- |
| ****1**** | **52,195** | **57,106** | **29,002** |
| ****2**** | **1,087,985** | **1,581,494** | **1,438,543** |
| ****3**** | **2,455,105** | **6,908,093** | **5,111,577** |
| ****#1AND #2 AND #3**** | **646** | **823** | **605** |

# Supplementary Material 1: Data selection, extraction, management, and analysis procedures.

We included European population-based studies with clearly defined sampling strategies or national/multinational school-based studies reporting the prevalence of myopia or providing sufficient data to estimate it. Eligible studies had to represent populations of any age from a European country or region and provide a clear definition of myopia, including spherical equivalent refractive error (SE) of ≤ -0.50 D. Given the limited data available, we extracted and analysed high myopia prevalence based on the definitions provided in the original studies. Only two studies reported the prevalence of high myopia, both using a cutoff of ≤ -5.00 D. Studies were excluded if they were clinical- or hospital-based surveys, myopia registries, self-reported near-sightedness, or lacked data on eligible participants. Studies focusing on specific populations that could not be generalised to the general population, studies with non-representative populations (e.g., studies limited to males, military populations, or non-random samples), or using visual acuity as a surrogate for refractive error were also excluded. Additionally, studies involving participants with serious medical conditions (e.g., leukemia, heart disorders, or syndromes associated with myopia) or eye disorders (e.g., congenital cataracts) were excluded. Studies focused exclusively on children undergoing myopia control treatments were not considered. Inclusion was restricted to European population-based studies with clearly defined sampling strategies or national/multinational school-based studies that reported the prevalence of myopia or provided sufficient data to compute the estimate.

The selection of studies was conducted independently by two reviewers (AM-R and CL). The initial search yielded several articles that were then subjected to a screening process, during which duplicates were removed. The screening was facilitated using Rayyan software, where both reviewers, blinded to each other's assessments, reviewed titles and abstracts. In instances where eligibility could not be ascertained from the abstract, the full texts of the studies were evaluated. The reviewers then independently assessed the full texts of studies identified as eligible. Disagreements were resolved by a third reviewer (AG).

Data extraction was carried out independently by AM-R and CL using a standardised form. The following information was collected: the country of origin, the participant recruitment period, the method of myopia diagnosis (cycloplegic or non-cycloplegic refraction), the myopia definition criteria, the number of participants with complete data, the participants' age (children aged 6−11 years and 12–17 years; adults aged 18–39 years and ≥ 40 years), sex distribution, and the prevalence of myopia and high myopia. Additionally, data on study design, site (population-based or school-based), recruitment location (urban/rural), sampling method, and enrollment rates were also recorded. To ensure the inclusion of all pertinent studies, a multilingual approach was employed. Specifically, articles published in languages other than English were reviewed using translation tools and by consulting bilingual experts. The authors were contacted when clarification was needed or when additional data were required. Disagreements or uncertainties among the authors were resolved through discussion.

The risk of bias for each study was assessed independently by AM-R and CL using the Joanna Briggs Institute Critical Appraisal Checklist for Studies Reporting Prevalence Data. Any discrepancies were resolved through discussion until a consensus was reached. Detailed quality information for each study is provided in Supplementary Figure 1. For each study, nine quality items were assessed using a "yes," "no," or "unclear" scale. 1) the suitability of the sample frame for the target population; 2) the appropriateness of the sampling method; 3) the adequacy of the sample size; 4) the clarity in the description of study subjects and setting; 5) the completeness of data analysis for the sample; 6) the validity of the diagnostic tool; 7) the reliability of the diagnostic system; 8) the appropriateness of statistical analysis; and 9) the adequacy of the response rate (> 70%). Each study was evaluated using information from associated citations and, when available, study protocols. While the quality assessment confirmed that all studies met the criteria for data extraction and synthesis, a sensitivity analysis was conducted for studies reporting outcomes using non-cycloplegic refraction instead of cycloplegic refraction.

# Supplementary Table 3: PRISMA 2020 Checklist.

| **Section and Topic** | **Item #** | **Checklist item** | **Location where item is reported** |
| --- | --- | --- | --- |
| **TITLE** | | |  |
| Title | 1 | Identify the report as a systematic review. | 1 |
| **ABSTRACT** | | |  |
| Abstract | 2 | See the PRISMA 2020 for Abstracts checklist. | 2 |
| **INTRODUCTION** | | |  |
| Rationale | 3 | Describe the rationale for the review in the context of existing knowledge. | 3 |
| Objectives | 4 | Provide an explicit statement of the objective(s) or question(s) the review addresses. | 4, 5 |
| **METHODS** | | |  |
| Eligibility criteria | 5 | Specify the inclusion and exclusion criteria for the review and how studies were grouped for the syntheses. | 5, 6 |
| Information sources | 6 | Specify all databases, registers, websites, organisations, reference lists and other sources searched or consulted to identify studies. Specify the date when each source was last searched or consulted. | 5 |
| Search strategy | 7 | Present the full search strategies for all databases, registers and websites, including any filters and limits used. | 5; Supplementary Table 1 |
| Selection process | 8 | Specify the methods used to decide whether a study met the inclusion criteria of the review, including how many reviewers screened each record and each report retrieved, whether they worked independently, and if applicable, details of automation tools used in the process. | 6, 7 |
| Data collection process | 9 | Specify the methods used to collect data from reports, including how many reviewers collected data from each report, whether they worked independently, any processes for obtaining or confirming data from study investigators, and if applicable, details of automation tools used in the process. | 6, 7 |
| Data items | 10a | List and define all outcomes for which data were sought. Specify whether all results that were compatible with each outcome domain in each study were sought (e.g. for all measures, time points, analyses), and if not, the methods used to decide which results to collect. | 6, 7 |
|  | 10b | List and define all other variables for which data were sought (e.g. participant and intervention characteristics, funding sources). Describe any assumptions made about any missing or unclear information. | 6, 7; Supplementary Material 1 |
| Study risk of bias assessment | 11 | Specify the methods used to assess risk of bias in the included studies, including details of the tool(s) used, how many reviewers assessed each study and whether they worked independently, and if applicable, details of automation tools used in the process. | 5, 6, 7; Supplementary Material 1; Figure 1 |
| Effect measures | 12 | Specify for each outcome the effect measure(s) (e.g. risk ratio, mean difference) used in the synthesis or presentation of results. | 7, 8, 9 |
| Synthesis methods | 13a | Describe the processes used to decide which studies were eligible for each synthesis (e.g. tabulating the study intervention characteristics and comparing against the planned groups for each synthesis (item #5)). | 7, 8, 9; Table 1 |
|  | 13b | Describe any methods required to prepare the data for presentation or synthesis, such as handling of missing summary statistics, or data conversions. | 7, 8, 9; Supplementary Material 1 |
|  | 13c | Describe any methods used to tabulate or visually display results of individual studies and syntheses. | 7, 8, 9; Supplementary Material 1 |
|  | 13d | Describe any methods used to synthesize results and provide a rationale for the choice(s). If meta-analysis was performed, describe the model(s), method(s) to identify the presence and extent of statistical heterogeneity, and software package(s) used. | 7, 8, 9; Supplementary Material 1 |
|  | 13e | Describe any methods used to explore possible causes of heterogeneity among study results (e.g. subgroup analysis, meta-regression). | 7, 8, 9; Supplementary Material 1 |
|  | 13f | Describe any sensitivity analyses conducted to assess robustness of the synthesized results. | 7, 8, 9; Supplementary Material 1 |
| Reporting bias assessment | 14 | Describe any methods used to assess risk of bias due to missing results in a synthesis (arising from reporting biases). | 7, 8, 9; Supplementary Material 1 |
| Certainty assessment | 15 | Describe any methods used to assess certainty (or confidence) in the body of evidence for an outcome. | 7, 8, 9 |
| **RESULTS** | | |  |
| Study selection | 16a | Describe the results of the search and selection process, from the number of records identified in the search to the number of studies included in the review, ideally using a flow diagram. | 9, 10; Figure 1 |
|  | 16b | Cite studies that might appear to meet the inclusion criteria, but which were excluded, and explain why they were excluded. | Not Applicable |
| Study characteristics | 17 | Cite each included study and present its characteristics. | 9, 10; Table 1 |
| Risk of bias in studies | 18 | Present assessments of risk of bias for each included study. | Supplementary Figure 1 |
| Results of individual studies | 19 | For all outcomes, present, for each study: (a) summary statistics for each group (where appropriate) and (b) an effect estimate and its precision (e.g. confidence/credible interval), ideally using structured tables or plots. | 9, 10; Table 1 |
| Results of syntheses | 20a | For each synthesis, briefly summarise the characteristics and risk of bias among contributing studies. | 9, 10, 11 |
|  | 20b | Present results of all statistical syntheses conducted. If meta-analysis was done, present for each the summary estimate and its precision (e.g. confidence/credible interval) and measures of statistical heterogeneity. If comparing groups, describe the direction of the effect. | 9, 10, 11; Table 1-3; Figure 3 |
|  | 20c | Present results of all investigations of possible causes of heterogeneity among study results. | 10, 11, 12, 13 |
|  | 20d | Present results of all sensitivity analyses conducted to assess the robustness of the synthesized results. | 10, 11, 12, 13 |
| Reporting biases | 21 | Present assessments of risk of bias due to missing results (arising from reporting biases) for each synthesis assessed. | 9; Supplementary Figure 1 |
| Certainty of evidence | 22 | Present assessments of certainty (or confidence) in the body of evidence for each outcome assessed. | 10, 11, 12, 13; Table 2-3; Figure 3 |
| **DISCUSSION** | | |  |
| Discussion | 23a | Provide a general interpretation of the results in the context of other evidence. | 13, 14, 15 |
|  | 23b | Discuss any limitations of the evidence included in the review. | 16, 17 |
|  | 23c | Discuss any limitations of the review processes used. | 16, 17 |
|  | 23d | Discuss implications of the results for practice, policy, and future research. | 17, 18 |
| **OTHER INFORMATION** | | |  |
| Registration and protocol | 24a | Provide registration information for the review, including register name and registration number, or state that the review was not registered. | 5 |
|  | 24b | Indicate where the review protocol can be accessed, or state that a protocol was not prepared. | 5 |
|  | 24c | Describe and explain any amendments to information provided at registration or in the protocol. | Not Applicable |
| Support | 25 | Describe sources of financial or non-financial support for the review, and the role of the funders or sponsors in the review. | 3, 18, 19 |
| Competing interests | 26 | Declare any competing interests of review authors. | 18 |
| Availability of data, code and other materials | 27 | Report which of the following are publicly available and where they can be found: template data collection forms; data extracted from included studies; data used for all analyses; analytic code; any other materials used in the review. | 18 |

From: Page MJ, McKenzie JE, Bossuyt PM, Boutron I, Hoffmann TC, Mulrow CD, Shamseer L, Tetzlaff JM, Akl EA, Brennan SE, Chou R, Glanville J, Grimshaw JM, Hróbjartsson A, Lalu MM, Li T, Loder EW, Mayo-Wilson E, McDonald S, McGuinness LA, Stewart LA, Thomas J, Tricco AC, Welch VA, Whiting P, Moher D. The PRISMA 2020 statement: an updated guideline for reporting systematic reviews. BMJ. 2021 Mar 29;372:n71. doi: 10.1136/bmj.n71. PMID: 33782057; PMCID: PMC8005924.

# Supplementary Table 4: MOOSE Checklist for Meta-analyses of Observational Studies.

| **Item No** | **Recommendation** | **Location where item is reported** |
| --- | --- | --- |
| **Reporting of background should include** | | |
| 1 | Problem definition | 4 |
| 2 | Hypothesis statement | Not Applicable |
| 3 | Description of study outcome(s) | 4, 5 |
| 4 | Type of exposure or intervention used | 4, 5 |
| 5 | Type of study designs used | 4, 5 |
| 6 | Study population | 4, 5 |
| **Reporting of search strategy should include** | | |
| 7 | Qualifications of searchers (e.g., librarians and investigators) | 5 |
| 8 | Search strategy, including time period included in the synthesis and key words | 5, 6; Supplementary Table 1; Supplementary Material 1 |
| 9 | Effort to include all available studies, including contact with authors | Supplementary Material 1 |
| 10 | Databases and registries searched | 5, 6; Supplementary Material 1 |
| 11 | Search software used, name and version, including special features used (eg, explosion) | 5, 6; Supplementary Material 1 |
| 12 | Use of hand searching (eg, reference lists of obtained articles) | 5, 6; Supplementary Material 1 |
| 13 | List of citations located and those excluded, including justification | Figure 1; Supplementary Table 2 |
| 14 | Method of addressing articles published in languages other than English | Supplementary Material 1 |
| 15 | Method of handling abstracts and unpublished studies | Supplementary Material 1 |
| 16 | Description of any contact with authors | Supplementary Material 1 |
| **Reporting of methods should include** | | |
| 17 | Description of relevance or appropriateness of studies assembled for assessing the hypothesis to be tested | 5, 6 |
| 18 | Rationale for the selection and coding of data (e.g., sound clinical principles or convenience) | 7, 8, 9 |
| 19 | Documentation of how data were classified and coded (e.g., multiple raters, blinding and interrater reliability) | 6, 7, 8, 9 |
| 20 | Assessment of confounding (e.g., comparability of cases and controls in studies where appropriate) | 5, 6, 7, 8, 9 |
| 21 | Assessment of study quality, including blinding of quality assessors, stratification or regression on possible predictors of study results | 5, 6, 7, 8, 9; Supplementary Material 1; Supplementary Figure 1 |
| 22 | Assessment of heterogeneity | 7, 8, 9 |
| 23 | Description of statistical methods (e.g., complete description of fixed or random effects models, justification of whether the chosen models account for predictors of study results, dose-response models, or cumulative meta-analysis) in sufficient detail to be replicated | 7, 8, 9 |
| 24 | Provision of appropriate tables and graphics | Supplementary Table 1; Supplementary Table 2 |
| **Reporting of results should include** | | |
| 25 | Graphic summarizing individual study estimates and overall estimate | Figure 3 |
| 26 | Table giving descriptive information for each study included | Table 1 |
| 27 | Results of sensitivity testing (e.g., subgroup analysis) | 9, 10, 11; Table 2; Table 3 |
| 28 | Indication of statistical uncertainty of findings | 9, 10, 11, 12, 13; Table 1; Table 2; Table 3; Figure 3 |

| **Reporting of discussion should include** | | |
| --- | --- | --- |
| 29 | Quantitative assessment of bias (e.g., publication bias) | 13, 14, 15 |
| 30 | Justification for exclusion (e.g., exclusion of non-English language citations) | Not Applicable |
| 31 | Assessment of quality of included studies | 15 |
| **Reporting of conclusions should include** | | |
| 32 | Consideration of alternative explanations for observed results | 15, 16, 17 |
| 33 | Generalization of the conclusions (ie, appropriate for the data presented and within the domain of the literature review) | 16, 17, 18 |
| 34 | Guidelines for future research | 17 |
| 35 | Disclosure of funding source | 3, 18, 19 |

From: Stroup DF, Berlin JA, Morton SC, Olkin I, Williamson GD, Rennie D, Moher D, Becker BJ, Sipe TA, Thacker SB. Meta-analysis of observational studies in epidemiology: a proposal for reporting. Meta-analysis Of Observational Studies in Epidemiology (MOOSE) group. JAMA. 2000 Apr 19;283(15):2008-12. doi: 10.1001/jama.283.15.2008. PMID: 10789670.

# Supplementary Figure 1: Summary of the quality assessment of 22 studies on the prevalence of myopia in Europe, conducted using the JBI Critical Appraisal Checklist.

|  | Armenia (Giloyan et al., 2017) | Bulgaria (Dragomirova et al. 2022) | Denmark (Lundberg et al., 2018) | Denmark (Hansen et al., 2020) | Finland (Aine, 1984) | Germany (Wolfram et al., 2014; Mirshahi et al., 2016) | Hungary (Németh et al., 2022) | Kazakhstan (Mukazhanova et al., 2022) | Norway (Midelfar et al., 2002) | Norway (Hagen et al., 2018) | Poland (Czepita et al., 2007; Czepita et al., 2019) | Republic of Ireland (O’Donoghue et al., 2010; French et al., 2012; McCullough et al., 2016) | Republic of Ireland (Harrington et al., 2019; Harrington et al., 2019) | Russia (Markova et al., 2021) | Russia (Bikbov et al., 2024) | Sweden (Villarreal et al., 2000) | The Netherlands (Tideman et al., 2018a; Tideman et al., 2016; Tideman et al., 2019) | The Netherlands (Tideman et al., 2018b) | The Netherlands (Enthoven et al., 2021) | United Kingdom (Logan et al., 2011) | United Kingdom (Sherwin et al., 2012) |
| --- | --- | --- | --- | --- | --- | --- | --- | --- | --- | --- | --- | --- | --- | --- | --- | --- | --- | --- | --- | --- | --- |
| **Suitability of the sample frame for the target population** | 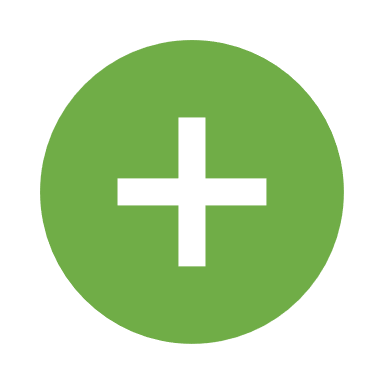 | 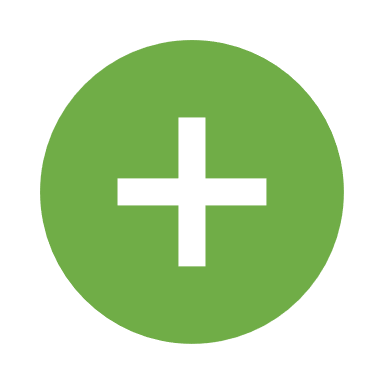 | 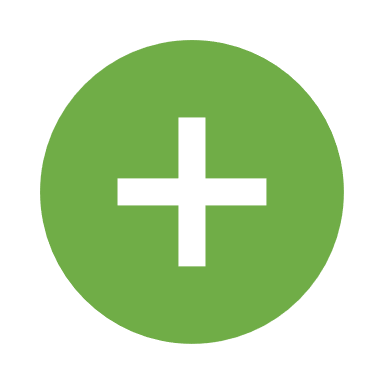 | 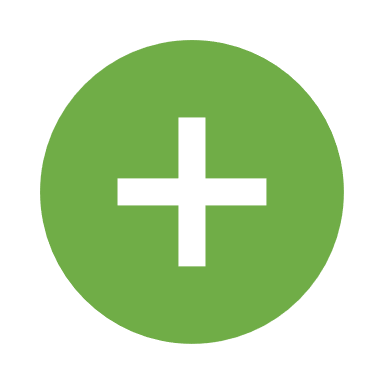 | 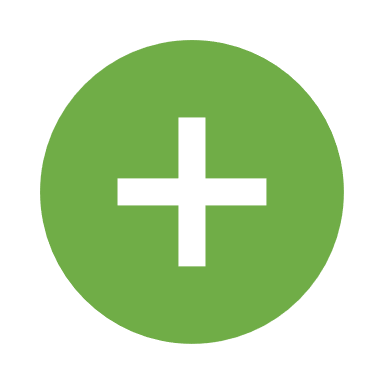 | 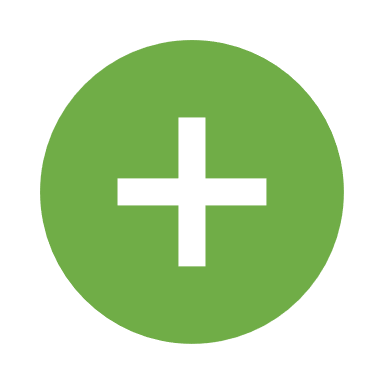 | 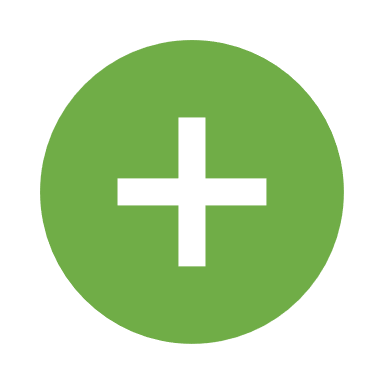 | 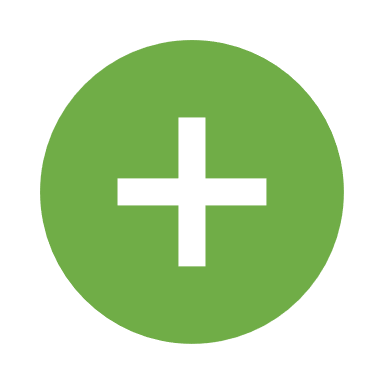 | 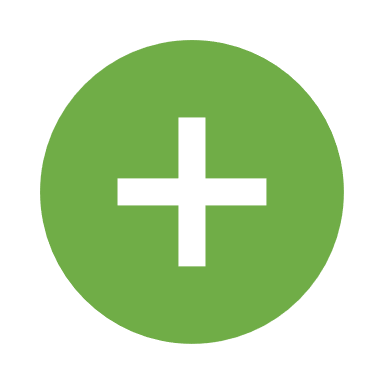 | 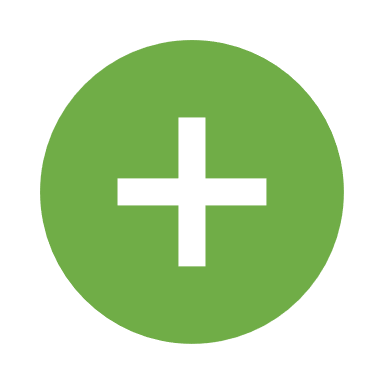 | 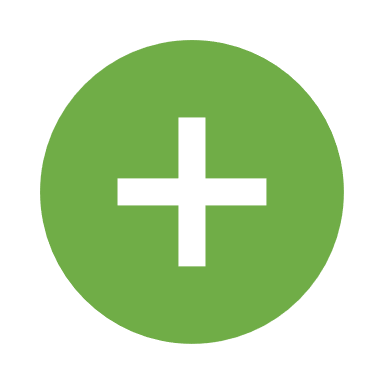 | 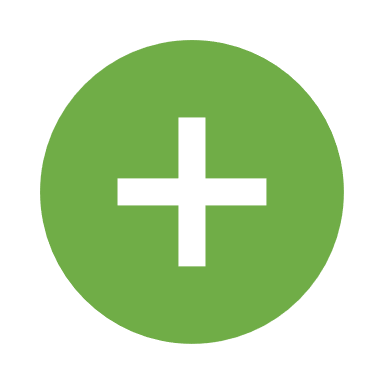 | 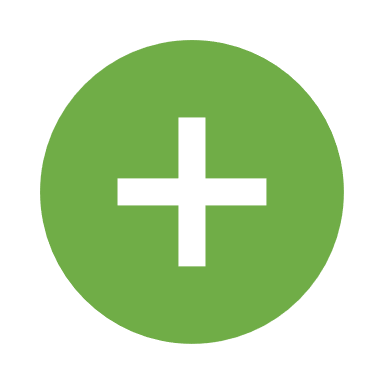 | 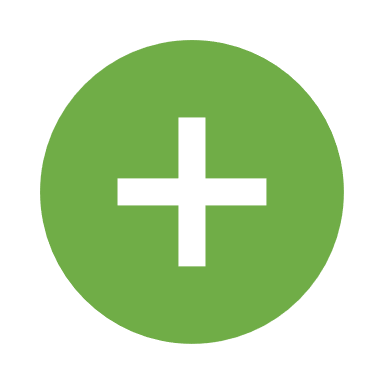 | 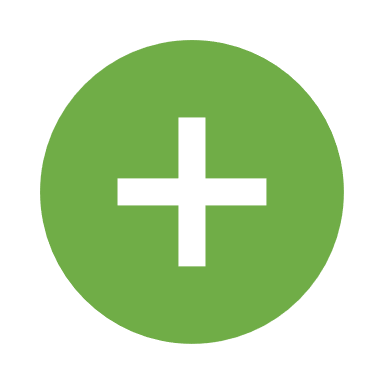 | 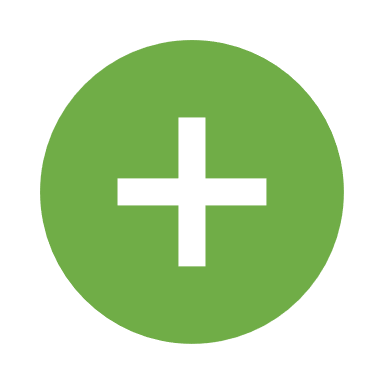 | 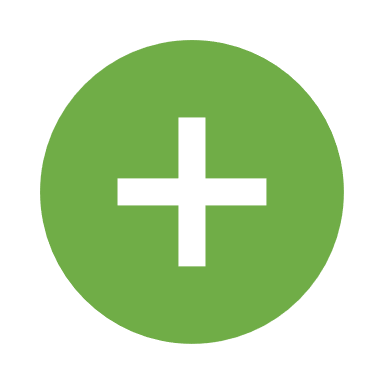 | 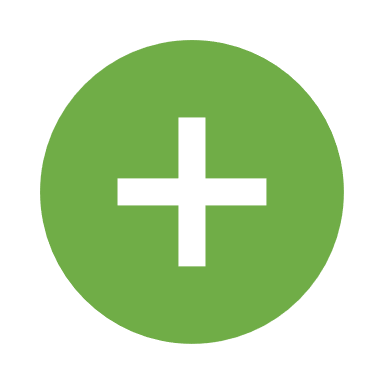 | 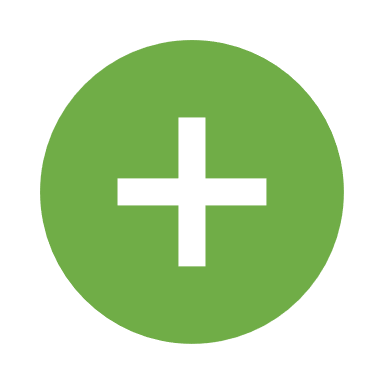 | 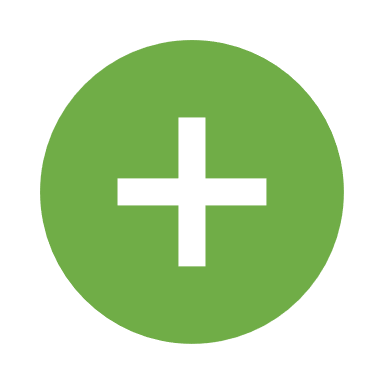 | 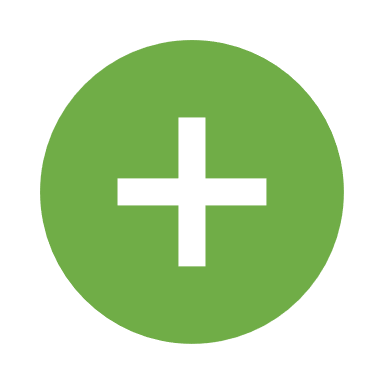 |
| **Appropriateness of the sampling method** | 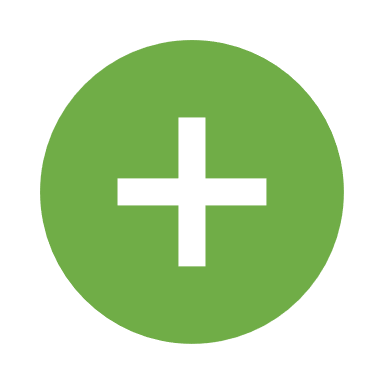 | 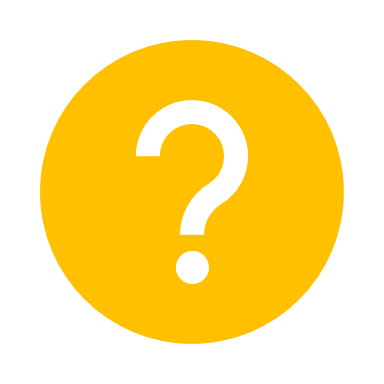 | 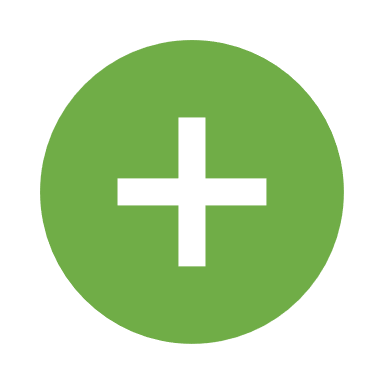 | 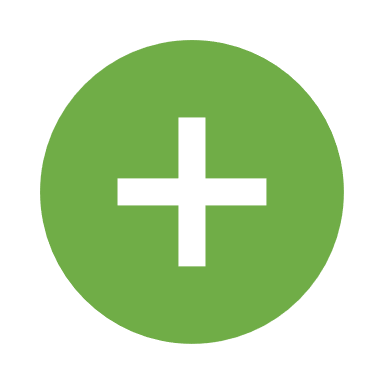 | 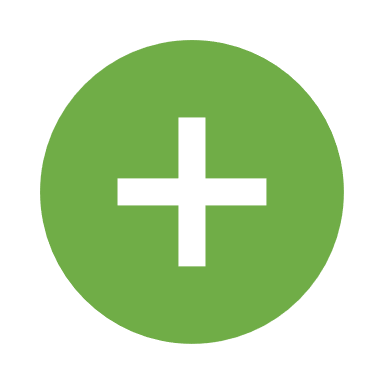 | 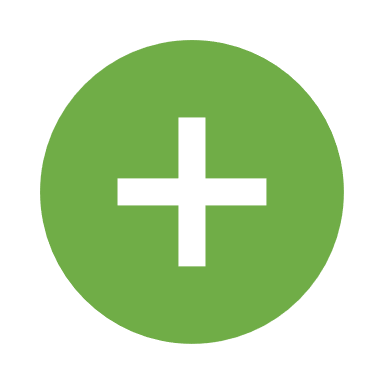 | 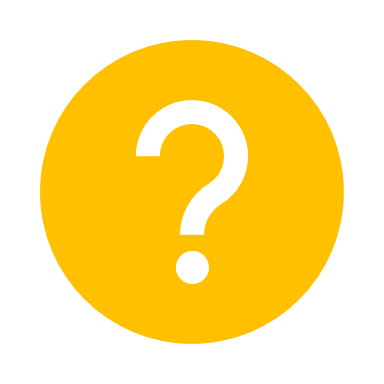 | 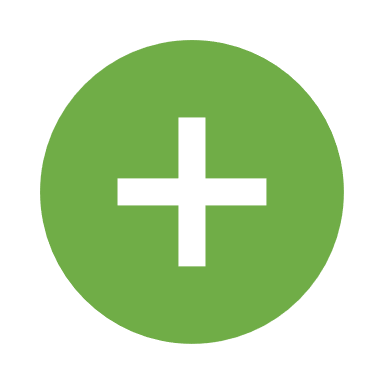 | 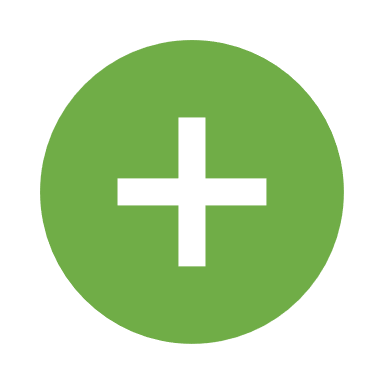 | 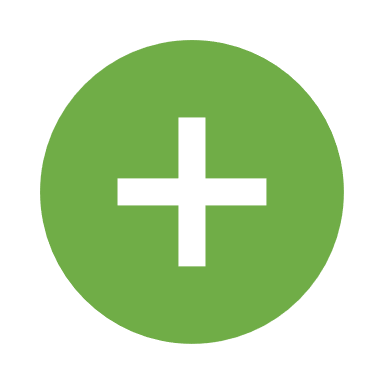 | 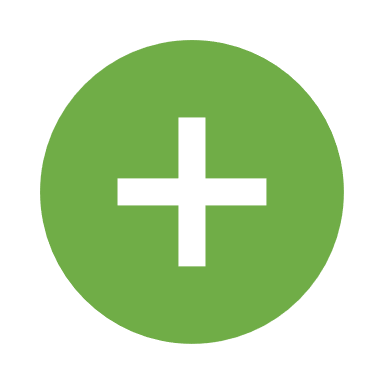 | 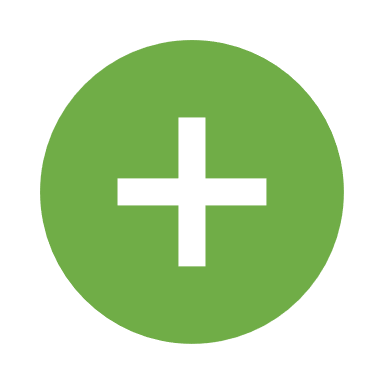 | 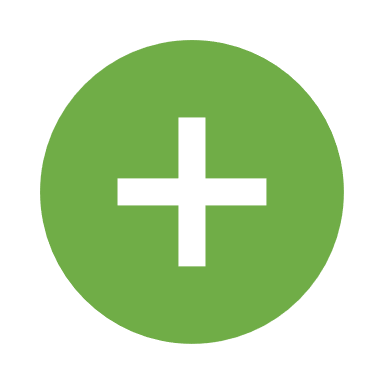 | 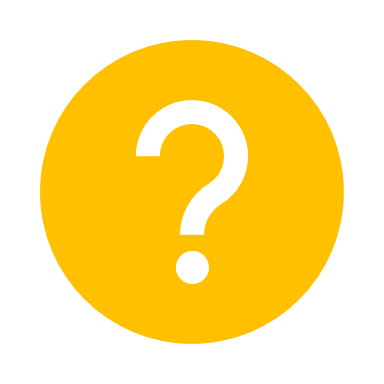 | 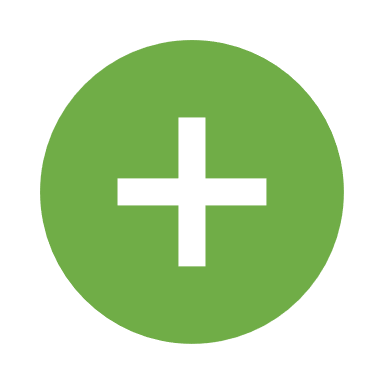 | 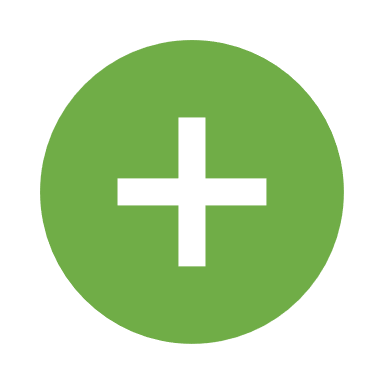 | 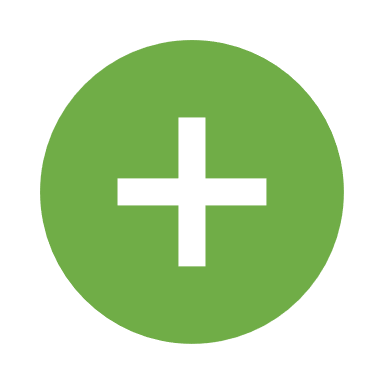 | 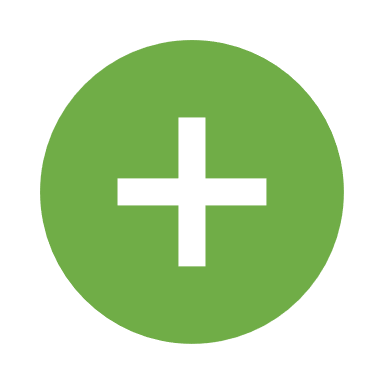 | 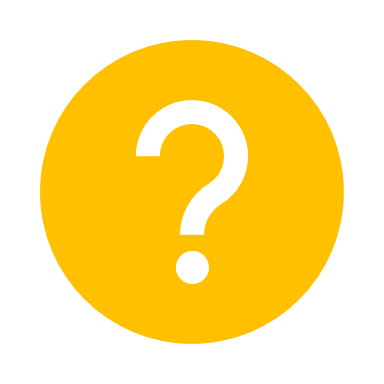 | 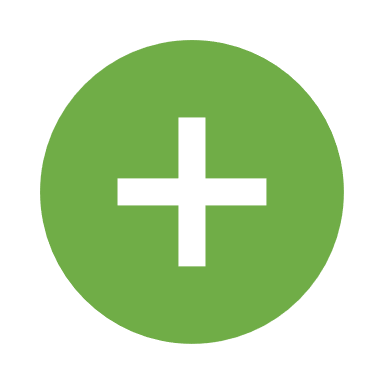 | 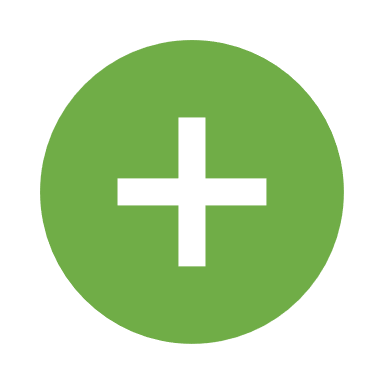 |
| **Adequacy of the sample size** | 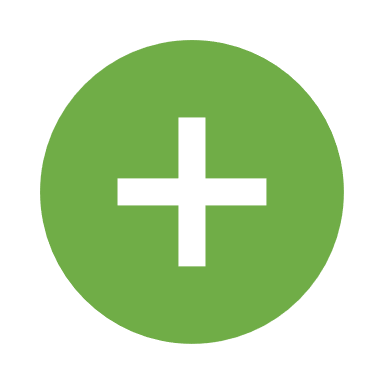 | 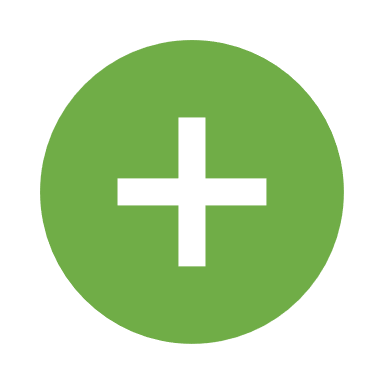 | 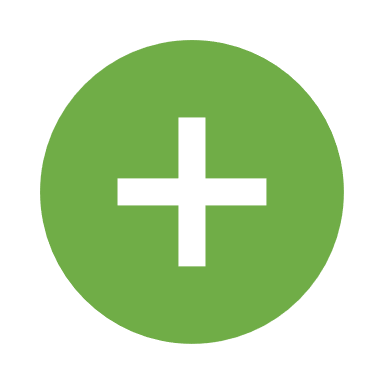 | 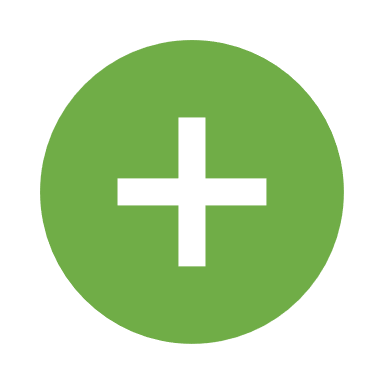 | 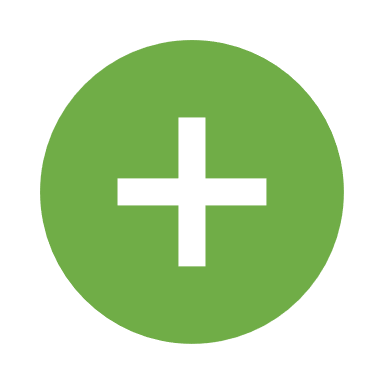 | 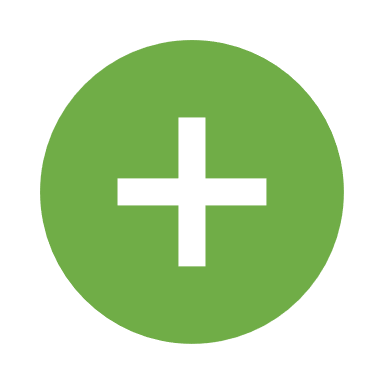 | 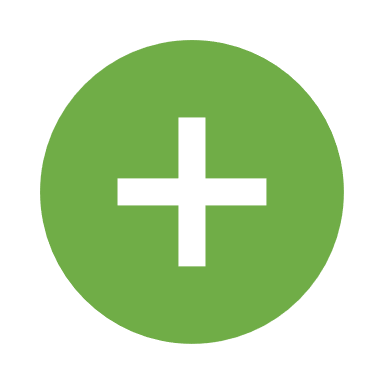 | 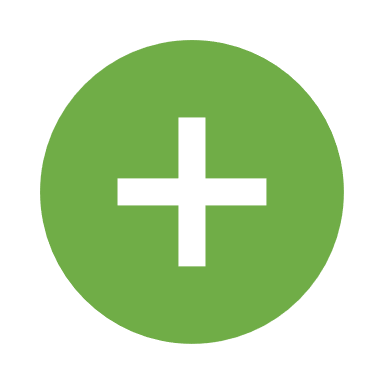 | 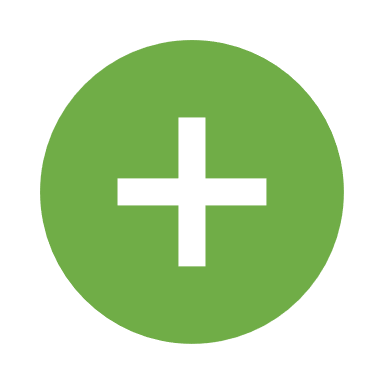 | 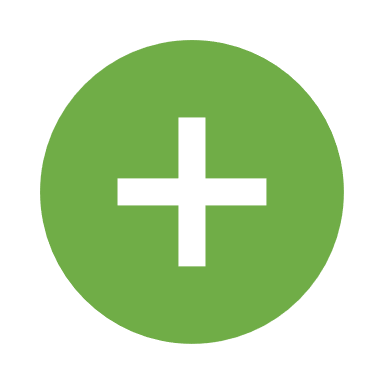 | 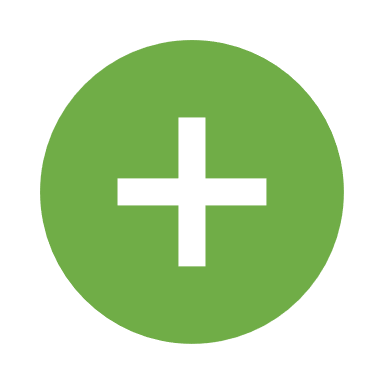 | 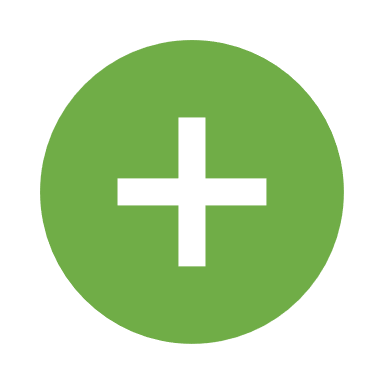 | 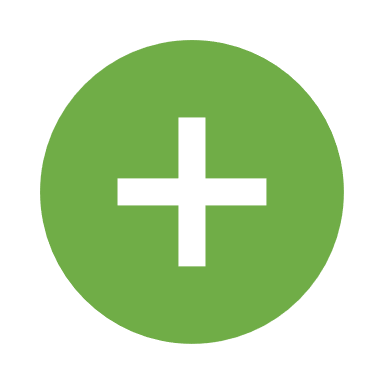 | 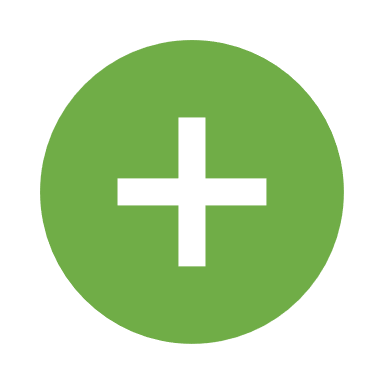 | 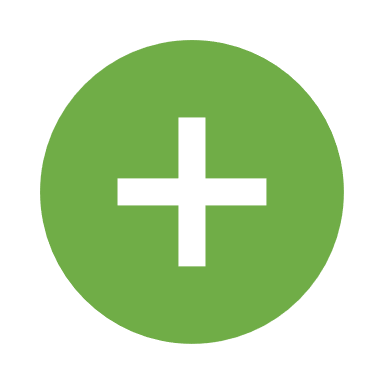 | 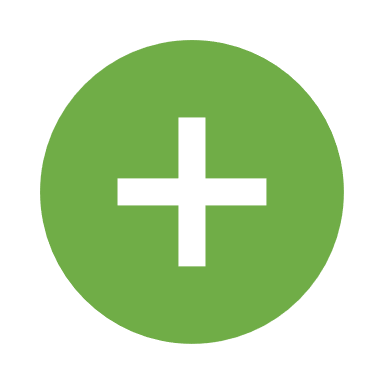 | 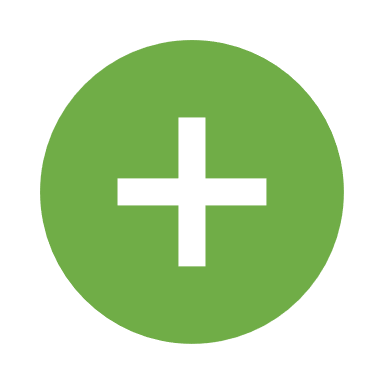 | 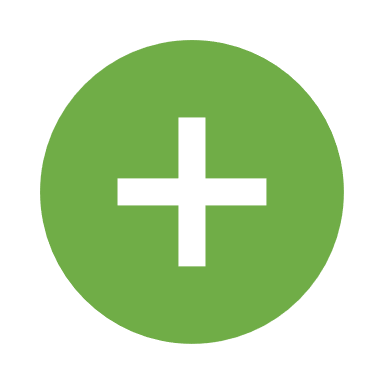 | 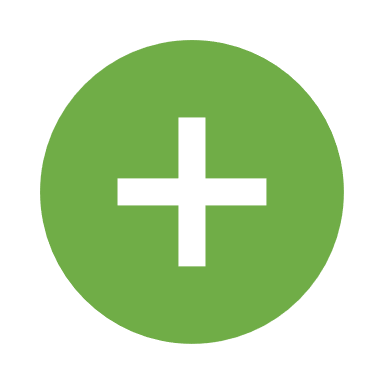 | 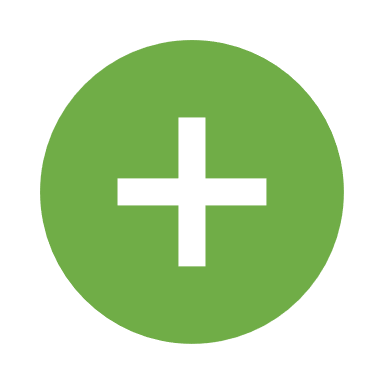 | 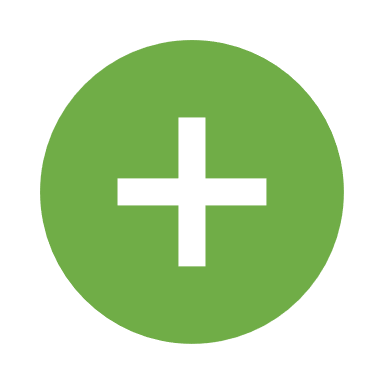 |
| **Detail in the description of subjects and setting** | 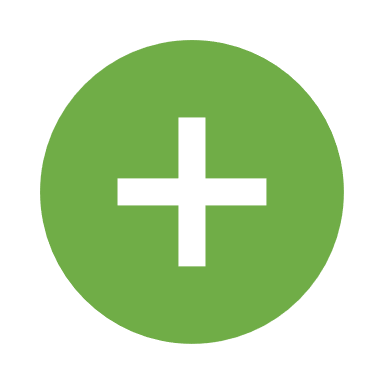 | 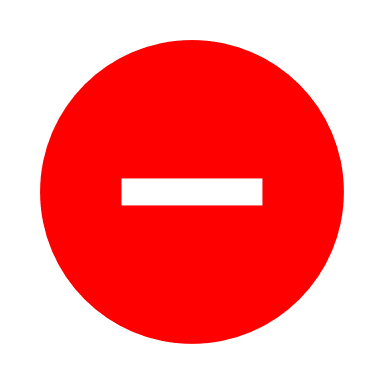 | 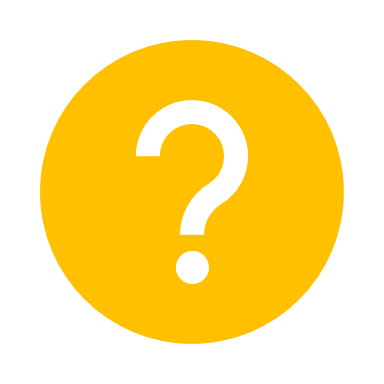 | 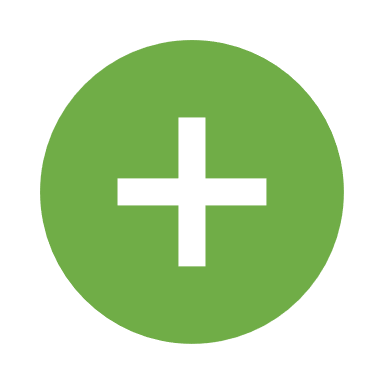 | 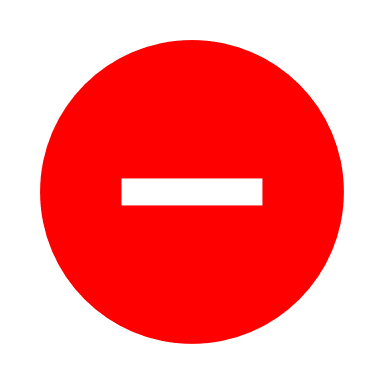 | 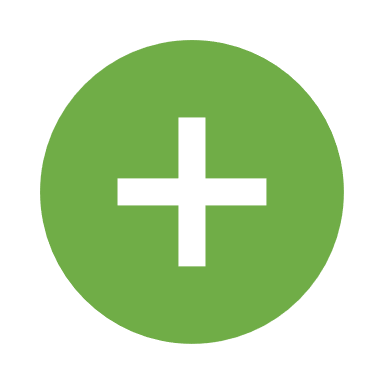 | 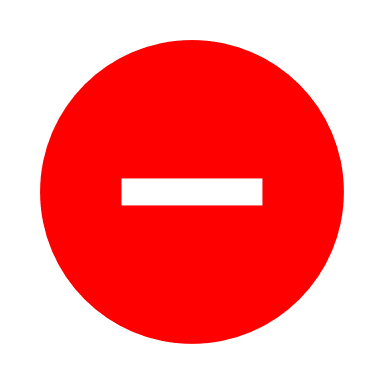 | 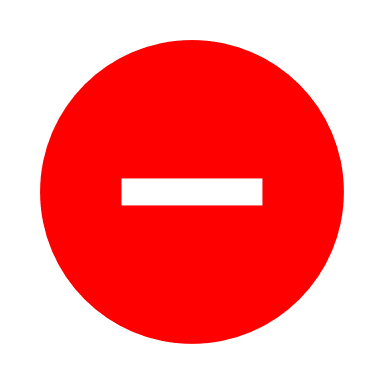 | 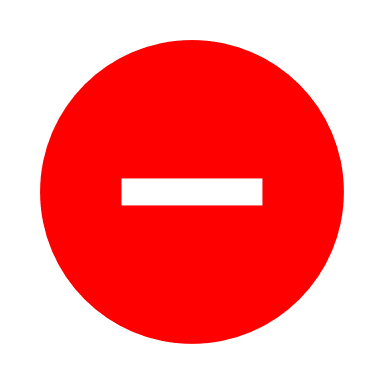 | 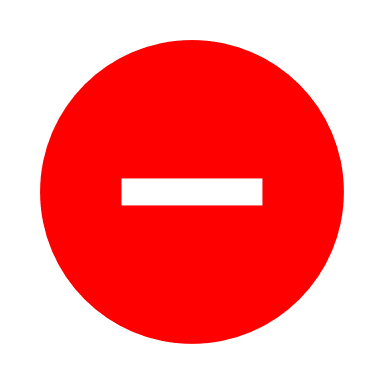 | 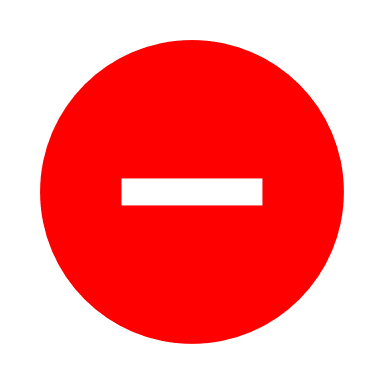 | 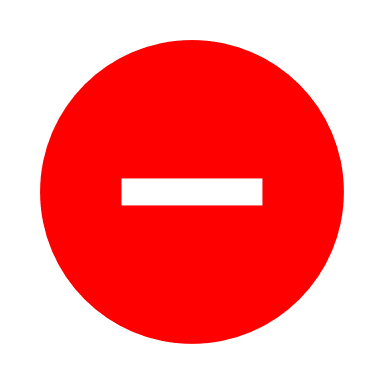 | 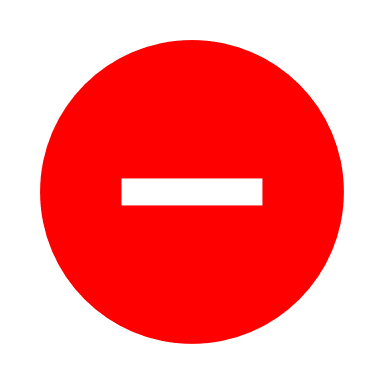 | 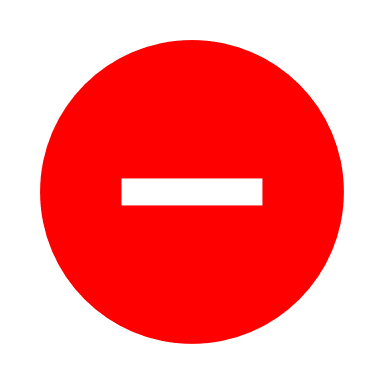 | 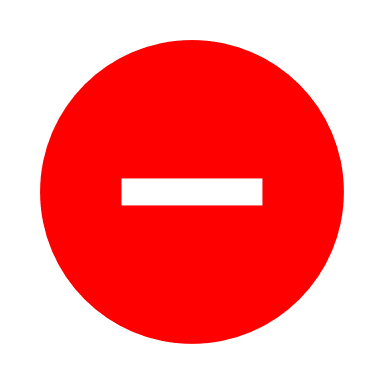 | 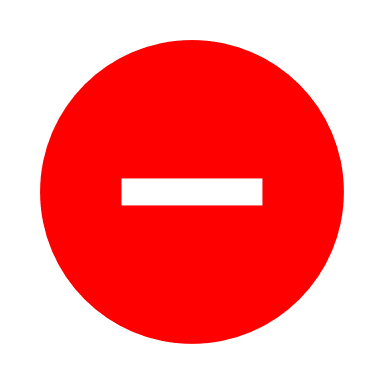 | 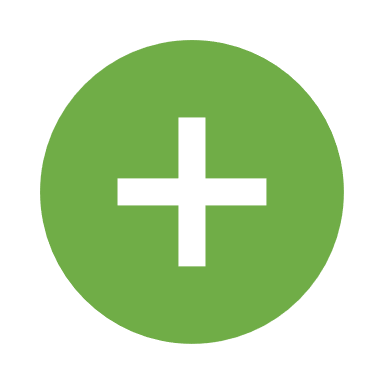 | 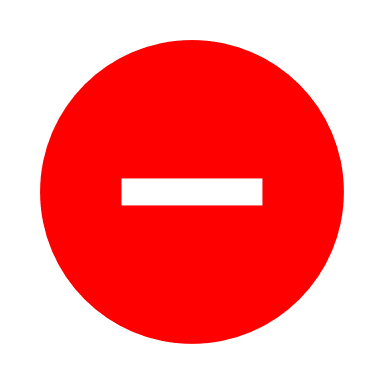 | 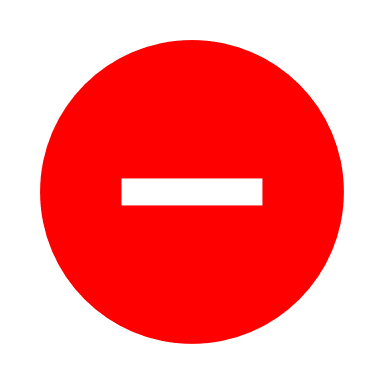 | 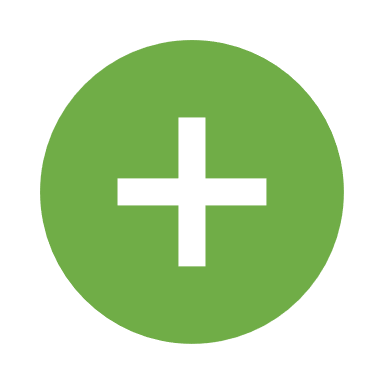 | 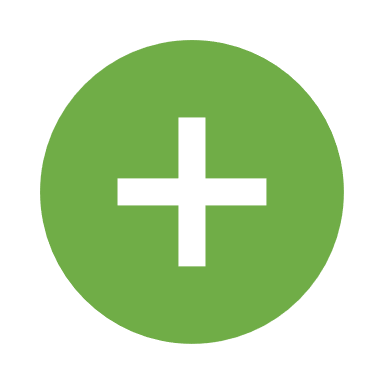 |
| **Completeness of data analysis for the sample** | 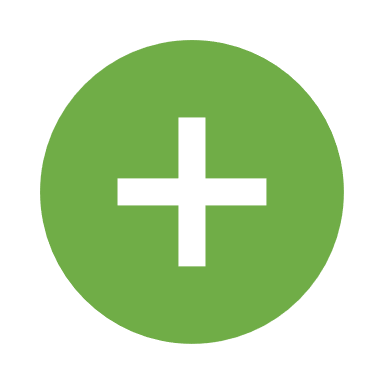 | 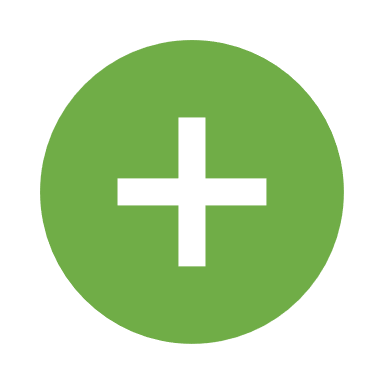 | 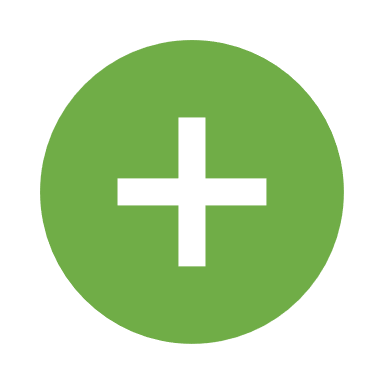 | 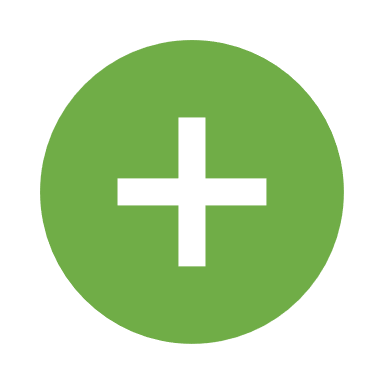 | 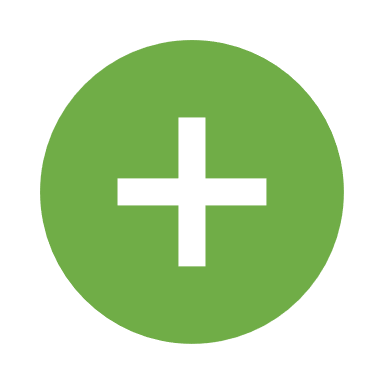 | 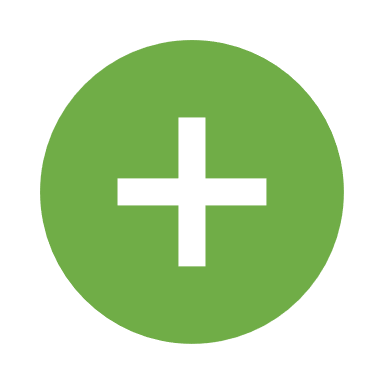 | 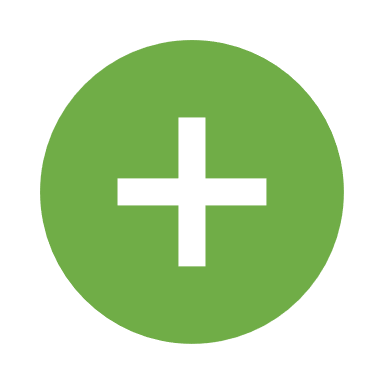 | 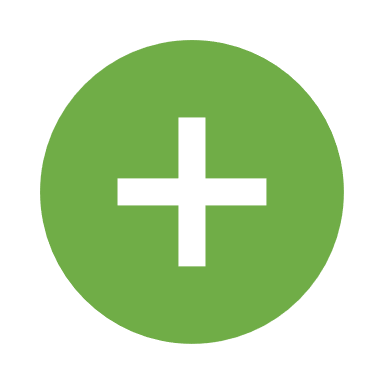 | 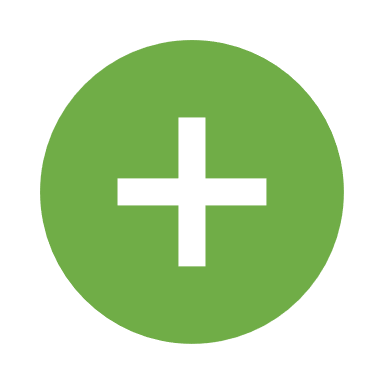 | 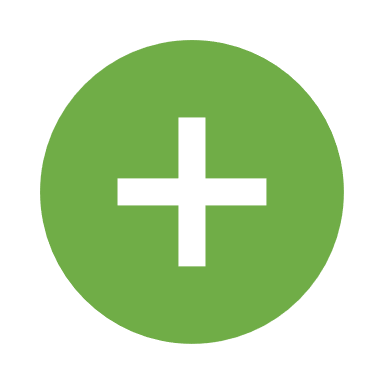 | 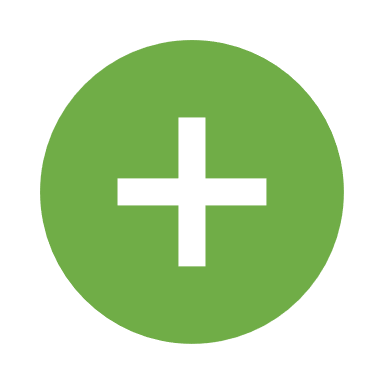 | 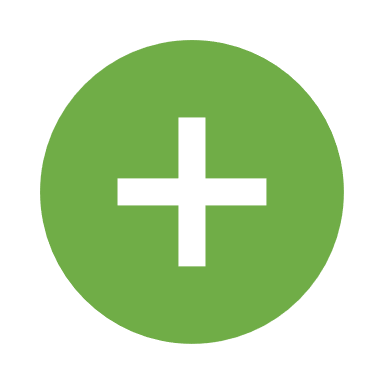 | 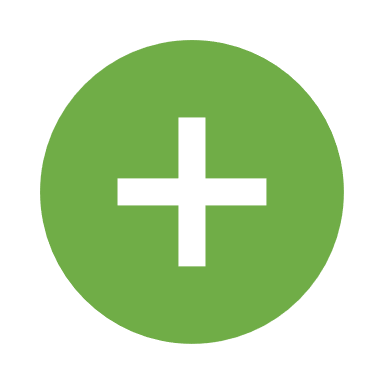 | 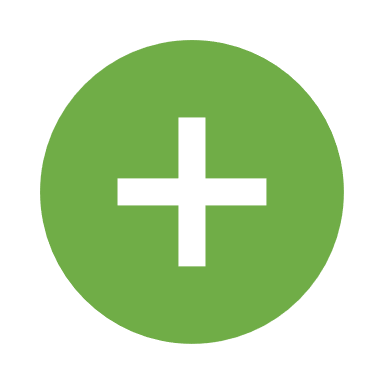 | 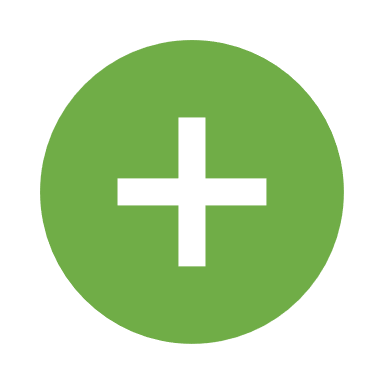 | 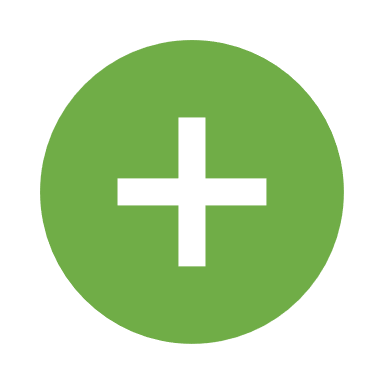 |  |  |  |  |  |
| **Validity of the diagnostic tool** |  |  |  |  |  |  |  |  |  |  |  |  |  |  |  |  |  |  |  |  |  |
| **Reliability of the diagnostic system** |  |  |  |  |  |  |  |  |  |  |  |  |  |  |  |  |  |  |  |  |  |
| **Appropriateness of the statistical analysis** |  |  |  |  |  |  |  |  |  |  |  |  |  |  |  |  |  |  |  |  |  |
| **Adequacy of the response rate and its management** |  |  |  |  |  |  |  |  |  |  |  |  |  |  |  |  |  |  |  |  |  |

# Supplementary Table 5: Prevalence of myopia by study and age group.

| Study characteristics | | | Prevalence | | | |
| --- | --- | --- | --- | --- | --- | --- |
| Country | **Citation(s)** | **Diagnostic method** | **6-11 years** | **12-17 years** | **18-39 years** | **+40 years** |
| Armenia | Giloyan et al., 2017 | Cycloplegic | - | - | - | - |
| Bulgaria | Dragomirova et al. 2022 | Non–Cycloplegic | - | - | - | - |
| Denmark | Lundberg et al., 2018 | Cycloplegic | - | - | - | - |
|  | Hansen et al., 2020 | Non–Cycloplegic | - | 24.9% | - | - |
| Finland | Aine, 1984 | Cycloplegic | - | - | 22.6% | - |
| Germany | Wolfram et al., 2014; Mirshahi et al., 2016 | Non–Cycloplegic | - | - | - | 19.6% |
| Hungary | Németh et al., 2022 | Non–Cycloplegic | - | - | 58.7% | - |
| Kazakhstan | Mukazhanova et al., 2022 | Cycloplegic | - | - | - | - |
| Norway | Midelfar et al., 2002 | Non–Cycloplegic | - | - | 35.0% | 30.3% |
|  | Hagen et al., 2018 | Non–Cycloplegic | - | 11.0% | - | - |
| Poland | Czepita et al., 2007; Czepita et al., 2019 | Cycloplegic | 7.8% | 19.1% | 32.6% | - |
| Republic of Ireland | O’Donoghue et al., 2010; French et al., 2012; McCullough et al., 2016 | Cycloplegic | 2.8% | 17.7% | 18.6% | - |
|  | Harrington et al., 2019; Harrington et al., 2019 | Cycloplegic | 3.7% | 22.8% | - | - |
| Russia | Markova et al., 2021 | NR | 20.1% | - | - | - |
|  | Bikbov et al., 2024 | Cycloplegic | - | - | - | - |
| Sweden | Villarreal et al., 2000 | Cycloplegic | - | 49.7% | - | - |
| The Netherlands | Tideman et al., 2018^a^; Tideman et al., 2016; Tideman et al., 2019 | Cycloplegic | 5.3% | - | - | - |
|  | Tideman et al., 2018^b^ | Non–Cycloplegic | - | - | - | 37.0% |
|  | Enthoven et al., 2021 | Cycloplegic | - | 18.9% | - | - |
| United Kingdom | Logan et al., 2011 | Cycloplegic | 9.5% | 29.4% | - | - |
|  | Sherwin et al., 2012 | Non–Cycloplegic | - | - | - | 27.8% |
|  | Cumberland et al., 2018 | Non–Cycloplegic | - | - | - | 47.0% |

# Supplementary Figure 2: Pooled prevalence of myopia in Europe by age groups (6–11, 12–17, and 18–39 years) stratified by study site (a) school-based and (b) population-based. Analysis includes only studies using cycloplegic refraction. GLMM (generalised linear mixed model); CI (confidence interval).

# Supplementary Figure 3: Funnel plot for the meta-analysis using the Freeman-Tukey double arcsine transformation. Egger's linear regression test p-value = 0.0183.

|  |  |  |  |  |  |  |  |  |  |  |  |  |  |  |  |
| --- | --- | --- | --- | --- | --- | --- | --- | --- | --- | --- | --- | --- | --- | --- | --- |

# Supplementary Figure 4: Funnel plot for the meta-analysis using the generalised linear mixed model (GLMM). Peters' regression test p-value = 0.0469.

# Supplementary Figure 5: Funnel plot for the meta-analysis of studies employing cycloplegic refraction, using the Freeman-Tukey double arcsine transformation. Egger's linear regression test p-value = 0.5073.

# Supplementary Figure 6: Funnel plot for the meta-analysis of studies employing cycloplegic refraction, using the generalised linear mixed model (GLMM). Peters' regression test p-value = 0.8960.
