# Supplementary material for: Prevalence of myopia in Europe: a systematic review and meta-analysis of data from 14 countries
Source: Lancet Reg Health Eur. 2025 May 22;54:101319. doi: 10.1016/j.lanepe.2025.101319 (PMC12266183; doi:10.1016/j.lanepe.2025.101319)
Supplement: Summary in Portuguese [file mmc2.docx]

Supplementary Summary in Portuguese

*This translation in Portuguese was submitted by the authors and we reproduced it as supplied. It has not been peer reviewed. Our editorial processes have only been applied to the original abstract in English, which should serve as reference for this manuscript.*

Prevalência de miopia na Europa: uma revisão sistemática e meta-análise de dados de 14 países

**Resumo
Contexto:** Embora a prevalência de miopia tenha aumentado nos países do Leste Asiático, o impacto da miopia na Europa é menos conhecido. Realizámos uma revisão sistemática e meta-análise para estimar a prevalência de miopia na Europa e nos países europeus.

**Métodos:** Foram pesquisadas as bases de dados PubMed, Scopus e Web of Science para identificar estudos sobre a prevalência de miopia disponíveis até janeiro de 2024, sem restrição de língua. Incluímos estudos europeus transversais e de coorte com estratégias de amostragem bem definidas e excluímos inquéritos clínicos, registos de miopia, miopia autorreportada e populações não representativas. A prevalência agregada foi estimada utilizando modelos de efeitos aleatórios. A heterogeneidade foi avaliada através do teste χ² aplicado à estatística Q de Cochran e quantificada pela estatística I². O protocolo do estudo foi pré-registado no PROSPERO (CRD42023471527).

**Resultados:** Foram triadas 2.074 publicações, das quais 22 estudos (de 14 países europeus; n = 128.012) foram incluídos nas meta-análises. A prevalência agregada de miopia foi de 23,5% (IC 95%: 18,5–29,3; I² = 99,7%), variando entre 11,9% na Finlândia e 49,7% na Suécia. Nos estudos que utilizaram refração cicloplégica, a prevalência de miopia foi de 18,9% (IC 95%: 13,2–26,5%; I² = 99,7%), em comparação com 31,2% (IC 95%: 24,9–38,3%; I² = 99,3%) nos que recorreram à refração não cicloplégica. As análises de subgrupos e de meta-regressão realizadas para identificar fontes de heterogeneidade evidenciaram uma prevalência significativamente mais baixa em crianças (6–11 anos; 5,5%) do que em adolescentes (12–17 anos; 25,2%) e adultos (18–39 anos; 24,3%) nos estudos com refração cicloplégica. Não foram observadas diferenças estatisticamente significativas entre sexos. Verificou-se um aumento significativo da prevalência de miopia entre os períodos de 2000–2010 e 2011–2022 (p = 0,040), embora as tendências específicas por faixa etária se tenham mantido relativamente estáveis.

**Interpretação:** A prevalência de miopia na Europa é inferior à observada na Ásia, apresentando um aumento menos pronunciado, que desaparece após estratificação por refração cicloplégica e idade. Estes resultados realçam a necessidade de dados específicos por faixa etária e refração cicloplégica em estudos futuros, a fim de reduzir a heterogeneidade. A representação desigual dos países incluídos nesta meta-análise pode limitar a generalização dos resultados.

**Financiamento:** Este estudo foi financiado pela Fundação para a Ciência e Tecnologia, através do CHRC.
